# Honeybee visitation to shared flowers increases *Vairimorpha ceranae* prevalence in bumblebees

**DOI:** 10.1002/ece3.10528

**Published:** 2023-09-20

**Authors:** Maryellen Zbrozek, Michelle L. Fearon, Chloe Weise, Elizabeth A. Tibbetts

**Affiliations:** ^1^ Department of Ecology & Evolutionary Biology University of Michigan Ann Arbor Michigan USA

**Keywords:** behavior, environmental transmission, host–parasite interactions, *Nosema*, parasite spillover

## Abstract

*Vairimorpha (=Nosema) ceranae* is a widespread pollinator parasite that commonly infects honeybees and wild pollinators, including bumblebees. Honeybees are highly competent *V. ceranae* hosts and previous work in experimental flight cages suggests *V. ceranae* can be transmitted during visitation to shared flowers. However, the relationship between floral visitation in the natural environment and the prevalence of *V. ceranae* among multiple bee species has not been explored. Here, we analyzed the number and duration of pollinator visits to particular components of squash flowers—including the petals, stamen, and nectary—at six farms in southeastern Michigan, USA. We also determined the prevalence of *V. ceranae* in honeybees and bumblebees at each site. Our results showed that more honeybee flower contacts and longer duration of contacts with pollen and nectar were linked with greater *V. ceranae* prevalence in bumblebees. Honeybee visitation patterns appear to have a disproportionately large impact on *V. ceranae* prevalence in bumblebees even though honeybees are not the most frequent flower visitors. Floral visitation by squash bees or other pollinators was not linked with *V. ceranae* prevalence in bumblebees. Further, *V. ceranae* prevalence in honeybees was unaffected by floral visitation behaviors by any pollinator species. These results suggest that honeybee visitation behaviors on shared floral resources may be an important contributor to increased *V. ceranae* spillover to bumblebees in the field. Understanding how *V. ceranae* prevalence is influenced by pollinator behavior in the shared floral landscape is critical for reducing parasite spillover into declining wild bee populations.

## Graphical abstract

We conducted an observational study among different pollinator communities in agricultural fields to understand (1) how floral visitation patterns differ among pollinator species and (2) whether the visitation patterns are linked with *V. ceranae* prevalence in honeybees and bumblebees. Our results showed that more honeybee flower contacts and longer duration of contacts with pollen and nectar simultaneously were linked with greater *V. ceranae* prevalence in bumblebees, even though honeybees were not the most frequent floral visitors. Meanwhile, *V. ceranae* prevalence in honeybees was unaffected by floral visitation behaviors by any pollinator species.

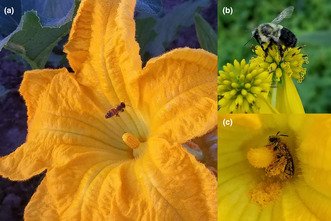

## 1 INTRODUCTION

Recent declines in wild and managed bee populations threaten the stability of pollination services that are vital for maintaining natural and agricultural ecosystems (Beismeijer et al., 2006, Potts et al., 2010). Several factors contribute to these declines, including the spread of multi‐host pathogens, habitat loss, and climate change (Burkle et al., 2013; Furst et al., 2014; Ricketts et al., 2008). Losses in pollinator community biodiversity and abundance lead to changes in flower visitation patterns (Albrecht et al., 2012; Beismeijer et al., 2006; Burkle et al., 2013), as well as changes in the risk of infectious disease within reduced pollinator communities (Fearon & Tibbetts, 2021; Figueroa et al., 2020; Graystock et al., 2020). Yet, it remains unclear how differences in floral visitation behaviors within pollinator communities affected by these declines may in turn affect the spread of pathogens.

Many pollinator pathogens and parasites (hereafter, “parasites”) are transmitted within and among species by visitation to flowers that were previously visited by infected bees (Durrer & Schmid‐Hempel, 1994; Graystock et al., 2015; Müller et al., 2019; Purkiss & Lach, 2019). The likelihood of parasite deposition and subsequent transmission on flowers depends on multiple factors, including flower traits, flower morphology, pollinator behavior, and the environment (Alger et al., 2019; Durrer & Schmid‐Hempel, 1994; Figueroa et al., 2019; Russell et al., 2019). Depending on the parasite, different plant components, including the floral tissue, pollen, and nectar, are implicated in transmission among pollinators (reviewed by McArt et al., 2014). In particular, differences in the rates of parasite deposition and acquisition of microorganisms on various flower parts may depend on how bees interact with the flowers during foraging visits. For example, bees foraging for pollen had greater rates of microbe deposition and acquisition on flowers than did bees foraging for nectar (Russell et al., 2019). However, pollinator visitation behaviors have been shown to have a complex relationship with the prevalence of bee parasites on flowers. In a study on pollinator viruses, flowers receiving longer visits were more likely to host viruses, but those with high visitation rates were less likely to host viruses (Alger et al., 2019). In a different study, *Crithidia bombi* survived longer when deposited inside the corolla rather than on the bract, but infection occurring from an encounter with the bract resulted in a more intense infection (Figueroa et al., 2019). Therefore, the ways in which infected bees interact with specific flower features and the duration and frequency of their visits will alter the likelihood of parasite deposition on floral surfaces and influence the probability of exposure and infection for later visitors. However, most studies on this topic have been conducted in the laboratory and have not fully considered the potential for parasite transmission via shared floral resources in natural settings.

Agricultural fields and the surrounding hedgerows may represent potential “hot spots” for parasite transmission within and among bee species on shared floral resources. Managed honeybees (*Apis mellifera*) are frequently brought to agricultural fields to provide pollination services, where they have ample opportunity to interact with wild pollinators that are also attracted to plentiful crop flowers or nearby hedgerows with wildflowers (Goulson & Hughes, 2015). The worldwide dispersal of *A. mellifera* (hereafter, “honeybees”) and its many parasites has consequently led to spillover (i.e., parasite transmission from reservoir populations to sympatric wildlife) to many naïve wild pollinators (Daszak et al., 2000; Goulson & Hughes, 2015; Keesing et al., 2006; Purkiss & Lach, 2019). Since honeybee colonies tend to send generalist foragers to a few flower patches at a time (Visscher & Seeley, 1982), it is possible that an infected colony may create localized floral hot spots where wild bees may acquire parasites. Increasingly, parasites previously thought to only infect honeybees are found in diverse populations of wild pollinators and seem to be contributing to their decline (Arbulo et al., 2015; Furst et al., 2014; Goulson & Hughes, 2015; Müller et al., 2019; Porrini et al., 2017; Purkiss & Lach, 2019).

One parasite of particular concern is the widely‐dispersed microsporidian parasite *Vairimorpha (*=*Nosema) ceranae* (Tokarev et al., 2020), which has been rapidly infecting honeybees and spilling over into wild bee populations over the past three decades (Chen et al., 2008; Fries, 2010; Paxton et al., 2007). Although *V. ceranae* is transmitted within honeybee hives through contaminated feces and pollen stores, transmission may also occur when bees encounter spores on contaminated flowers (Higes et al., 2010; Higes, Martín‐Hernández, Garrido‐Bailón, et al., 2008). Graystock et al. (2015) demonstrated that multiple pollinator parasites, including *V. ceranae*, can be effectively dispersed onto flowers by competent hosts and then vectored from flowers back to colonies by other pollinator species. Additionally, *V. ceranae* spores have been detected on the flowers of at least 14 plant genera in the field (Graystock et al., 2020). Therefore, contamination of shared floral resources is a likely mode of transmission for *V. ceranae* between different pollinator species, with dispersal potentially occurring through defecation on floral surfaces or through the rubbing off of spores that were attached to the bee cuticle (Bodden et al., 2019; Graystock et al., 2015; Piot et al., 2020). Furthermore, Graystock et al. (2015) found that *V. ceranae* transmission was very rapid in small experimental flight cages, but they recognized that whether parasite dispersal is similar in nature will depend on the characteristics of pollinator communities and environmental conditions. Despite clear experimental evidence for *V. ceranae* transmission on flowers, the relationship between specific pollinator visitation patterns and *V. ceranae* prevalence across managed and wild pollinator species in the field has remained understudied.

Here, we examine whether the prevalence of *V. ceranae* in managed and wild bee populations is influenced by the floral visitation behaviors of bees in the natural environment. We conducted an observational study of *V. ceranae* in honeybee (*A. mellifera*) and bumblebee (*Bombus* spp.) populations among different pollinator communities attracted to squash (Cucurbita) flowers to understand how floral visitation patterns differ among pollinator species and whether the visitation patterns are linked with *V. ceranae* prevalence in both host species. Specifically, we investigated how *V. ceranae* prevalence is linked with the number of generalist honeybee and bumblebee, specialist squash bee (*Eucera pruinosa*), and other pollinator taxa visits to flowers and the time each bee species spent interacting with different parts of the flowers during each visit. We hypothesized that higher numbers of visits and longer visits by potentially infected bees would increase the likelihood of *V. ceranae* transmission and correlate with higher *V. ceranae* prevalence. These findings will be important for determining the pollinator visitation behaviors that contribute the most to *V. ceranae* exposure and subsequent infection in honeybees and bumblebees as well as helping to establish whether *V. ceranae* transmission on flowers occurs under field conditions.

## 2 METHODS

### 2.1 Study system

*Vairimorpha ceranae* is a microsporidian parasite with a nearly global distribution. It was initially discovered in *Apis ceranae* and later spilled over into *A. mellifera* honeybees, where it appears to be more virulent than closely related parasites such as *V. apis* (Paxton et al., 2007). Recent studies have shown that wild native bees are also infected with *V. ceranae*, including many wild bumblebees (*Bombus* spp.), stingless bees (*Tetragonula hockingsi*, *Tetragonisca* spp., *Scaptotrigona* spp., *Melipona* spp.), and solitary bees (*Osmia bicornis*; Cilia et al., 2022; Furst et al., 2014; Graystock et al., 2013; Müller et al., 2019; Plischuk et al., 2009; Purkiss & Lach, 2019; Salvarrey et al., 2021). Transmission of *V. ceranae* between individuals is primarily fecal–oral or oral–oral, as it is spread through ingestion of contaminated food or contact with the feces of diseased hosts (Chen et al., 2008; Smith, 2012). In groups of honeybees, density‐dependent transmission may occur wherein higher ratios of individual‐to‐susceptible individuals result in higher proportions of susceptible individuals becoming infected, and higher transmission rates occur when the infected individuals are drones rather than workers (Roberts & Hughes, 2015). *Vairimorpha ceranae* germinates in the midgut of the bee, where the spore count can reach over 30 million, and it is then excreted as feces (Chen et al., 2008; Higes, Martín‐Hernández, Botías, et al., 2008; Paxton et al., 2007), potentially contributing very large numbers of spores to the environment (e.g., on floral surfaces). Symptoms of infection (nosemosis) in honeybees include digestive disorders, shortened life spans, atypical breeding behavior, reduced sucrose sensitivity, and diminished honey production; however, colony infection is often asymptomatic until sharp depopulation occurs, often in autumn and winter (Chen et al., 2008; Graystock et al., 2013; Higes et al., 2010; Higes, Martín‐Hernández, Botías, et al., 2008). Symptoms are generally assumed to be the same for wild bees, but data on this are limited aside from a few reports that *V. ceranae* may cause reduced survival, learning impairment, lower sucrose sensitivity, and cellular immunosuppression in bumblebees and stingless bees (Graystock et al., 2013; Macías‐Macías et al., 2020; Piiroinen & Goulson, 2016). Furthermore, *V. ceranae* infections suppress the pollinator immune response, which can lead to coinfection with other pathogens or parasites and an increased likelihood of mortality (Antúnez et al., 2009). The drastic effects of *V. ceranae* on pollinator health have been linked to the sudden collapse of honeybee colonies (Higes, Martín‐Hernández, Botías, et al., 2008) and may be an important factor in the recent declines of some wild bees (Furst et al., 2014; Goulson & Hughes, 2015; Graystock et al., 2013). In the United States, treatment and control for nosemosis over the past 60 years has primarily been through the use of fumagillin, which is banned across most of Europe (Martín‐Hernández et al., 2018). Although fumagillin suppresses microsporidia reproduction, it may actually increase the prevalence of *V. ceranae* over time alongside the decrease in its concentration after application (Huang et al., 2013). Other treatments such as nutritional supplements are used by beekeepers, but neither these nor fumagillin are effective in reducing *Vairimorpha* prevalence or intensity (Prouty et al., 2023).

This study was conducted in winter squash (Cucurbita) fields, which is a growing model system to study pollinators and their diseases (Fearon et al., 2023; Fearon & Tibbetts, 2021; Jones et al., 2021, 2022). Curcubita has large flowers that open for a single morning, approximately for 6 h (Nepi & Pacini, 1993). Male and female flowers occur on the same plant, with male flowers opening approximately half an hour earlier than female flowers (Nepi & Pacini, 1993). Female flowers only provide nectar rewards to visitors, whereas male flowers provide both pollen and nectar rewards and are more abundant in the fields. Early in the morning, pollinators visit male flowers with a high frequency and collect pollen, and later they are more attracted to female flowers which have more concentrated nectar, allowing for some pollen to be deposited during nectar foraging (Nepi & Pacini, 1993). Therefore, we focused our observations of pollinator visitation on the male flowers, where more types of interactions with the flower could occur.

**FIGURE 1:**
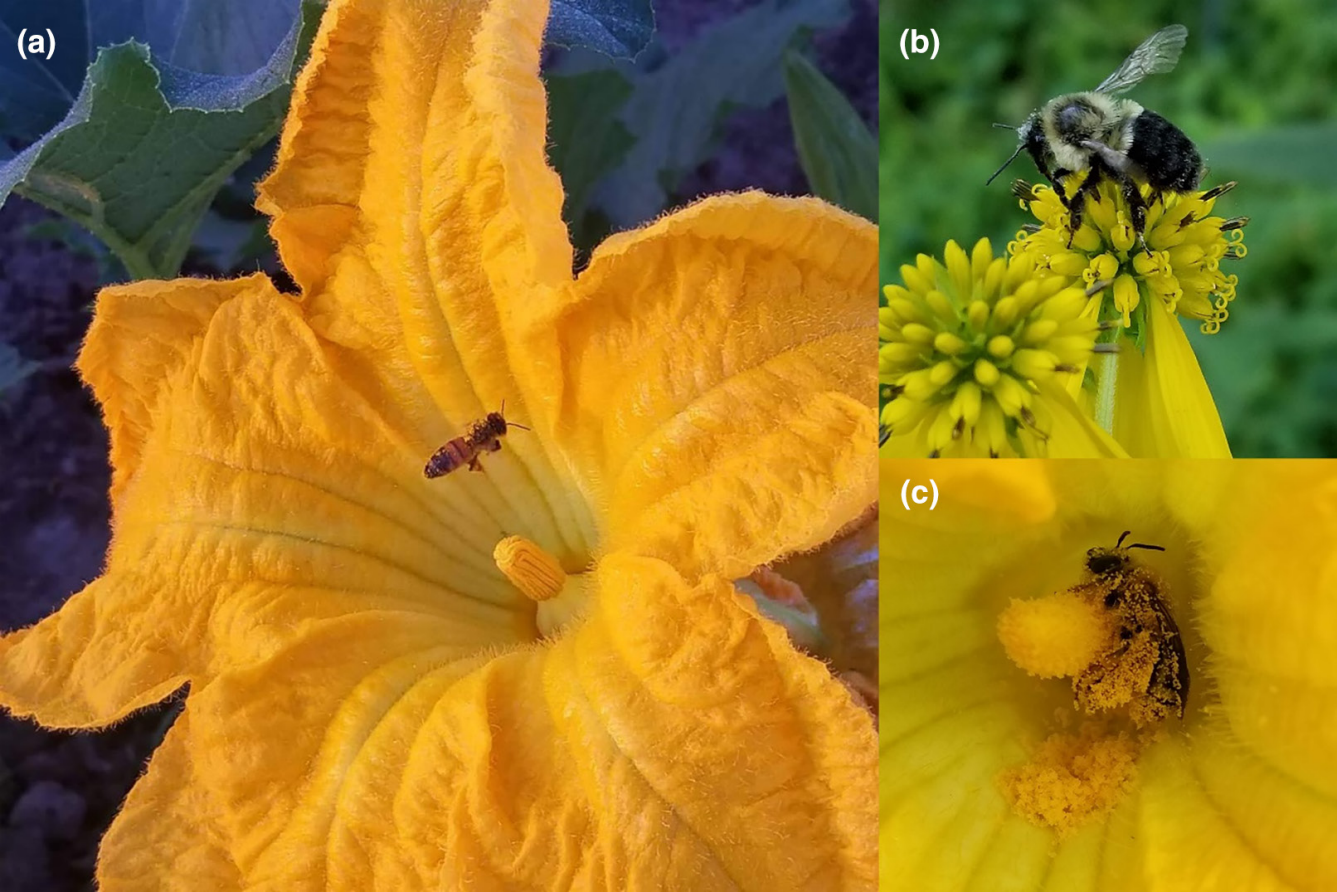
(a) Honeybee (*Apis mellifera*) visiting a male squash flower. (b) Eastern bumblebee (*Bombus impatiens*) foraging on wingstem (*Verbesina alternifolia*). (c) Female hoary squash bee (*Eucera pruinosa*), a squash pollen specialist, covered in pollen at the center of a male squash flower. All photos were taken by Michelle L. Fearon.

Squash flowers are commonly visited by generalist honeybees and bumblebees, among many other species (Figure 1a,b; López‐Uribe et al., 2016; Shuler et al., 2005). In particular, *Eucera pruinosa* (hereafter, squash bee) is a squash pollen specialist that depends nearly entirely on Cucurbita for pollen, is highly effective at pollinating Cucurbita plants, and can be highly abundant in some squash fields (Figure 1c; Hurd et al., 1971; Tepedino, 1981). Currently, there is little evidence that squash bees become infected by *V. ceranae*; detection of *V. ceranae* in *E. pruinosa* is very low (Jones et al., 2022), although high spore intensity has recently been observed for the first time in another *Eucera* species, *E. fervens* (Fernandez de Landa et al., 2022). However, non‐host pollinators can vector pollinator parasites among flowers and contribute to parasite spread (Davis et al., 2021). Considering the high attractiveness of squash flowers to many species of pollinators including both generalists and specialists, their short and specific period of flower opening, and their large size allowing relatively easy observation of pollinator behavior, squash flowers are both a likely ‘hot spot’ for transmission of pollinator diseases and an ideal system in which to study this possibility.

### 2.2 Sampling pollinators in the field

*Apis mellifera* and *Bombus* spp. (hereafter, “honeybee” and “bumblebee”, respectively) samples were collected from six winter squash farms in southeastern Michigan, USA (Appendix S1: Table S1) during two visits to each site between July 26 and August 30, 2016 during the peak squash bloom. The pollinator sampling described here includes a subset of the sites that were previously sampled in Fearon and Tibbetts (2021) and Fearon et al. (2023). In this study, we focus on *V. ceranae* prevalence in honeybees and bumblebees, while the prior studies examined links between the pollinator community composition and bee viral prevalence. Sites were at least 10 km apart to ensure that the pollinator communities were isolated from each other (Greenleaf et al., 2007). We only sampled on sunny days with windspeeds less than 2 m/s. To collect the bees, four 50 m transects were randomly placed at each field site. Three transects were placed in the field along the crop rows, while the fourth transect was placed along a field edge to sample bees foraging near native flowers and invasive weeds. All honeybees and bumblebees observed along the transect lines were collected using handheld nets or pan traps. Details on the trapping methods are included in Appendix S2 and Fearon and Tibbetts (2021).

All pollinator samples were stored on dry ice in the field and later placed in a −80°C freezer to maintain the integrity of the DNA for detection of *V. ceranae* presence. All bee species were identified using the Discover Life key (Ascher & Pickering, 2013). The collected bumblebee species were primarily *Bombus impatiens*, but also included *Bombus auricomus*, *Bombus bimaculatus*, *Bombus fervidus*, *Bombus griseocollis*, *Bombus pensylvanicus*, *Bombus sandersoni*, and *Bombus vagans* at very low densities across all sites (<4 non‐*B. impatiens* individuals total per site visit; Appendix S1: Table S2). *Apis mellifera* and *B. impatiens* were common at all six field sites. *Eucera pruinosa* squash bees were detected at all sites except the K site, but their abundance varied substantially among visits to the same site.

### 2.3 Collecting flower visitation data

During each visit to the six farms, we took 30‐min video recordings of pollinators visiting eight randomly selected male squash flowers per site (*N* = 112, mean video length: 30.87 min [sd: 3.75 min]). Each video was recorded between 07:30 AM and 12:00 PM on sunny, non‐windy days because all squash flowers opened at dawn and closed by 13:00 PM. Video recordings were watched to record data on the identity and frequency of pollinator visitors to the flowers. Pollinators captured on video were identified to genus where possible (e.g., *Apis*, *Bombus*, *Eucera*), or morphospecies for species that require close inspection and/or a key for accurate identification (Appendix S1: Table S3). Honeybees, bumblebees, and squash bees were easy to identify in the video recordings due to their relatively large body size and distinctive coloration. The behaviors of all other pollinators observed, including small green and olive halictids (e.g., *Augochlora*, *Augochlorella*, *Augochloropsis*, *Halictus*, and *Lasioglossum* genera), *Melissodes* spp., *Triepeolus* spp., *Vespula* wasp spp., and hoverflies, were grouped together into an “other pollinators” category to compare to honeybee, bumblebee, and squash bee behaviors in later analyses (see Statistics section).

During each individual pollinator's visit to the observed flower, we recorded the duration (seconds) of each visitor's interactions with specific flower parts, including petals (petal‐only), nectar (nectar‐only), pollen (pollen‐only), and both pollen and nectar simultaneously (pollen + nectar). Typically, large‐bodied bees, including honeybees and bumblebees, could not avoid contacting the stamen while drinking nectar (pollen + nectar), and led to relatively few observations of nectar‐only interactions with flowers (Appendix S1: Table S4). For this reason, the nectar‐only interactions were not considered as a substantial interaction type and were not included as a response variable in our main analyses. For each flower observed, the total duration of all types of interactions was summed for each pollinator group (honeybees, bumblebees, squash bees, or all other pollinators) and then divided by the number of flower visits for the respective pollinator group to generate the duration spent per visit by each pollinator group to each flower. Finally, to test how each pollinator group's visitation behavior impacted *V. ceranae* prevalence, we averaged the calculated visitation metrics for all flowers observed during the same site visit for each pollinator group. We followed the same process to calculate the average duration per visit of time spent on petal‐only, pollen‐only, and pollen + nectar interactions for each pollinator group. The number of visits for each pollinator group was the raw count of each type of pollinator that visited each observed flower within the 30‐min observation period, which was then averaged for each of the two visits to each site.

Evaluating the average duration bees spent per floral visit ensured that the duration metrics accurately reflected the time bees spent interacting with flowers without being skewed by the number of bee visitors. Each additional bee visitor inherently increased the total duration of time bees spent on flowers (*r* = .76, *t* = 11.52, df = 95, *p* < .001) but did not necessarily increase the duration per visit time (*r* = .02, *t* = 0.21, df = 95, *p* = .84). We predicted that bees that spent a greater amount of time per visit interacting with flowers would have a greater likelihood of either depositing or picking up *V. ceranae* spores on flowers and thus would have higher *V. ceranae* prevalence.

### 2.4 Detecting *V. ceranae* presence

Approximately eight honeybee and eight bumblebee individuals per visit to each field site were randomly selected to test for the presence or absence of *V. ceranae* (initial target *N* per species per site = 16). When less than our initial target of eight individuals of each species per visit were collected during a visit to a site (i.e., *n* < 16 individuals per species per site), then all individuals collected were tested for *V. ceranae* presence (Appendix S1: Table S5). Due to the inconsistent abundance of honeybees and bumblebees across sites and visits, we selected a minimum threshold of seven individual bees per species per site (regardless of visit) that could be standardized across all sites (total honeybee, *n* = 75; bumblebee, *n* = 86; Appendix S1: Table S5). Therefore, we tested between seven and 16 honeybees per site and between 10 and 16 bumblebees per site.

The selected bumblebees were predominantly *Bombus impatiens*, but also included single individuals from *Bombus fervidus*, *Bombus bimaculatus*, and *Bombus pensylvanicus* species that were all collected from a single field site visit that had relatively low *Bombus impatiens* abundance (Site E, Visit 1; Appendix S1: Table S2). Ultimately, we modeled the binary presence or absence of *V. ceranae* in individual honeybees and bumblebees and all sites had a minimum of seven samples tested for *V. ceranae* presence per host species. Given that different bumblebee species can have different disease transmission dynamics on flowers (Ruiz‐González et al., 2012), we confirmed that the few bumblebee individuals that were not *Bombus impatiens* did not alter the patterns we observed, as excluding those individuals did not change the results.

Abdominal contents were dissected from each sample using sterilized forceps and immediately placed on dry ice. Half of the abdomen was placed in a microcentrifuge tube for DNA analysis, and the other half was stored for reference. DNA was extracted using the DNeasy Blood & Tissue Kit (Qiagen, Germantown, MD, USA) following the manufacturer's instructions for tissue samples, and negative controls were included. Following extraction, DNA purity and concentration were quantified using Nanodrop 2000 software (Thermo Fisher Scientific, Waltham, MA, USA). One sample with a nucleic acid concentration of less than 10 ng/μL was removed from the study due to insufficient DNA extraction (a honeybee from Site E, Visit 1).

To ensure adequate extraction of bee DNA, polymerase chain reactions (PCR) were conducted on all samples using *A. mellifera* 18S rRNA gene primers, which produced bands at 784 bp (Cardinal et al., 2010) for both *A. mellifera* and *Bombus* spp. (Fearon & Tibbetts, 2021). Sequences for these bands were confirmed via Sanger sequencing (GenBank Accession No.: bee 18S rRNA, OQ545564–OQ545565). To determine presence or absence of *V. ceranae* in each sample, PCR was conducted with *V. ceranae*‐positive and H_2_O negative controls using the primers Nosema‐F (5′‐CGGATAAAAGAGTCCGTTACC‐3′) and Nosema‐R (5′‐TGAGCAGGGTTCTAGGGAT‐3′) for the *V. ceranae* large subunit ribosomal RNA gene (GenBank Accession No.: DQ486027; Chen et al., 2008). Details on the PCR procedure can be found in Appendix S2. A subset of samples was selected for Sanger sequencing to confirm the identification of *V. ceranae* (GenBank Accession No.: *V. ceranae* large subunit rRNA, OQ550096–OQ550100).

### 2.5 Statistical analysis

Analyses were performed using the statistical program R (version 4.2.1; R Core Team, 2020). First, we evaluated how pollinator visitation behavior varied among honeybees, bumblebees, squash bees, and other pollinators with a separate model for the following response variables: (1) number of visits to flowers per 30‐min video observation period, (2) total duration per visit of pollinator visits to flowers (seconds/visit), (3) duration pollinators spent on petals‐only per visit (seconds/visit), and (4) duration pollinators spent on pollen‐only per visit (seconds/visit), and (5) duration pollinators spent simultaneously on pollen + nectar per visit (seconds/visit; model output in Appendix S1: Table S6). Each model was a zero‐inflated generalized linear mixed effects model (GLMM) using a negative binomial distribution with a log link function and pollinator group (honeybees, bumblebees, squash bees, and other pollinators) as the main predictor for both the zero‐inflated and GLMM portions of the model (*glmmTMB* package; Brooks et al., 2017). The pollinator visitation data were aggregated by each flower observed for each pollinator group; therefore, a nested random effect of Flower ID (eight flowers/visit/site) within a visit to a site (two visits/site) within site (six sites) was used in each model. To model the duration per visit, we used the duration of behaviors in seconds as the response variable with an offset of the log of the number of pollinator visits +1 to correct for flowers with zero visits. We removed one outlier point from the number of visits per 30‐min data, where other pollinators visited a single flower over twice as many times as the next most visited flower in our study. Each model was checked for overdispersion, zero‐inflation, and spatial autocorrelation; none of these tests were significant (*DHARMa* package; Hartig, 2020). Then we followed up each model with a post hoc test to evaluate significant differences among honeybees, bumblebees, squash bees, and other pollinators' visitation behaviors (Appendix S1: Table S7; *emmeans* package; Lenth et al., 2020).

To evaluate *V. ceranae* prevalence in honeybees and bumblebees, we initially calculated the total apparent *V. ceranae* prevalence in each host species (*epiR* package; Stevenson et al., 2021) and used a Chi‐squared test of two proportions to determine if there was a significant difference among the two host species. We also tested for site‐level differences in *V. ceranae* prevalence in honeybees and bumblebees with GLMMs with a binomial distribution and visit to each site as a random effect. Then we ran two sets of models: the first to evaluate how the number of visits to flowers (per 30 min) at each site influenced *V. ceranae* prevalence in honeybees and bumblebees, and the second to test how the duration per visit of specific behaviors on the flowers was correlated with *V. ceranae* prevalence in each host species. All models were generalized linear mixed effects models with *V. ceranae* prevalence in either honeybees or bumblebees as the response variable, a binomial distribution, and a logit link function (package *lme4*; Bates et al., 2015). For all models, we used a random effect of each visit to a site nested within site to account for both the variation between the two different dates on which each site was visited and the variation among different sites, though the random effects were singular for many models (model outputs in Appendix S1: Tables S8 and S9). In the first set, models included the average number of honeybee visits, bumblebee visits, squash bee visits, and all other pollinator taxa visits to flowers during the 30‐min observation period as main effects. In the second set, we ran a series of models to evaluate the average total duration per visit (seconds/visit) and the durations per visit of petal‐only, pollen‐only, and pollen + nectar interactions of honeybees, bumblebees, squash bees, and all other pollinators that visited the flowers. To deal with zeros in the data, all main effects had “1” added to the value before log transforming the variable, and then they were scaled and centered to generate standardized estimates from the models. The variance inflation factor (VIF) for all main effects in all models was <4.7, indicating that there was no multicollinearity in our models. Additionally, none of the models were over‐dispersed. There was no evidence of spatial autocorrelation in the model residuals, indicating that *V. ceranae* prevalence was not correlated among sites based on their spatial proximity (*DHARMa* package; Hartig, 2020). Finally, we used a Bonferroni Correction of four comparisons to adjust our alpha significance threshold from 0.05 to 0.0125 to account for four separate analyses, one for each of four different pollinator duration behavioral parameters (total duration per visit, duration on petals per visit, duration on pollen per visit, duration on pollen + nectar per visit). Since the pollinator duration per visit on petals, pollen, and pollen + nectar were all nested within the total duration per visit, we used the Bonferroni Correction to reduce the risk of Type I errors (false‐positive). However, the number of visits model was not included in the Bonferroni Correction because it was the initial, planned comparison. Additionally, the number of pollinator visits and duration per visit on flowers were not correlated (*r* = .02, *t* = 0.021, df = 95, *p* = .84) and therefore unlikely to increase the likelihood of a Type I error. The number of visits model was evaluated with the usual 0.05 alpha threshold.

## 3 RESULTS

**FIGURE 2:**
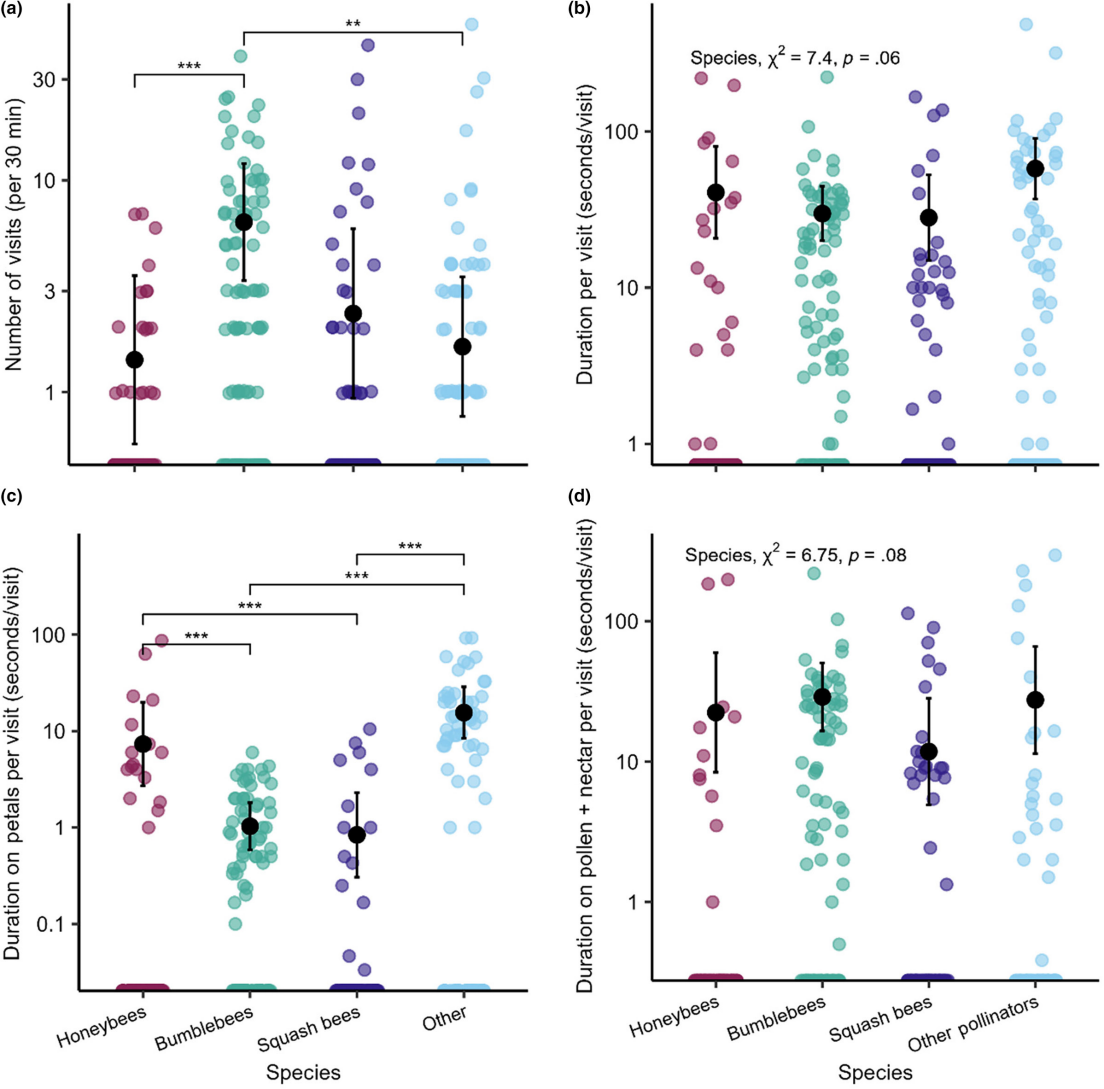
Honeybees and other pollinators had fewer visits to flowers compared to bumblebees, and honeybees and other pollinators spent more time on petals than bumblebees and squash bees. Total duration per visit and duration on pollen + nectar per visit did not differ among pollinator species. (a) Number of visits observed per 30 min by pollinator species, (b) total duration per visit (seconds/visit) by pollinator species, (c) duration on petals per visit (seconds/visit), (d) duration on pollen + nectar per visit (seconds/visit). Y axes are on a log scale, where zero values are on the x axis. Colored points are the raw data per flower observed, and the black points are the model‐predicted marginal means with 95% confidence intervals. Significant differences are indicated by the number of stars for each pair (Appendix S1: Table S7).

The number of pollinator visits by the four pollinator groups (honeybee, bumblebee, squash bees, and other pollinators) varied considerably among observed flowers (ranges: honeybees = 0–7, bumblebees = 0–38, squash bees = 0–43, other = 0–55; χ^2^ = 22.79, df = 3, *p* < .0001). Bumblebees had substantially more visits compared to honeybees (*p* = .0008) and combined other pollinators (0.0016), but there were no differences in the number of visits among honeybees, squash bees, and other pollinators (Figure 2a; Appendix S1: Tables S4, S6, and S7). Notably, honeybees were a relatively infrequent floral visitor. On the contrary, the time bees spent on flowers per visit (seconds) did not differ among honeybees, bumblebees, squash bees, and other pollinators (Figure 2b; χ^2^ = 7.39, df = 3, *p* = .06), despite substantial variation in total duration per visit among flowers observed (ranges: honeybees = 0–219.25, bumblebees = 0–222.75, squash bees = 0–166.5, other = 0–484 s). We further explored how bee species may differ in how much time per visit they spend interacting with different aspects of the flower, including the petals, pollen, and simultaneously contacting the pollen and nectar (pollen + nectar). Bumblebees and squash bees spent less time per visit on petals compared to honeybees (both *p* = .0001) or other pollinators (both *p* < .0001; Figure 2c). Other pollinators spent more time per visit on pollen‐only visits relative to honeybees (*p* < .0001), bumblebees (*p* < .0001), and squash bees (*p* = .0005; Appendix S1: Figure S1). On average, all four pollinator groups spent similar amounts of time per visit in contact with pollen + nectar (Figure 2d; χ^2^ = 6.75, df = 3, *p* = .08), though there was a wide range of visit times for pollen + nectar (ranges: honeybees = 0–197.75, bumblebees = 0–218.75, other = 0–295 s). Overall, each pollinator group differed in the number of visits and duration of time spent per visit interacting with different aspects of the flowers, which could contribute to variation in the likelihood of bees depositing or picking up parasite spores during floral visits.

*Vairimorpha ceranae* was highly prevalent in both honeybees and bumblebees at all six field sites. In total, 68.0% (95% CI: 56.7%–77.9%) of honeybees and 64.0% (95% CI: 52.9%–73.6%) of bumblebees had *V. ceranae* detected in their midguts. *V. ceranae* prevalence did not significantly differ between host species (*χ* ^2^ = 0.14, df = 1, *p* = .71). Among different sites, *V. ceranae* prevalence was consistently high in honeybees, from 57.1% to 81.3% (χ^2^ = 1.85, df = 5, *p* = .87), but *V. ceranae* prevalence did vary significantly in bumblebees from 40.0% to 93.8% (χ^2^ = 11.93, df = 5, *p* = .036, Appendix S1: Figure S2, Table S10).

**FIGURE 3:**
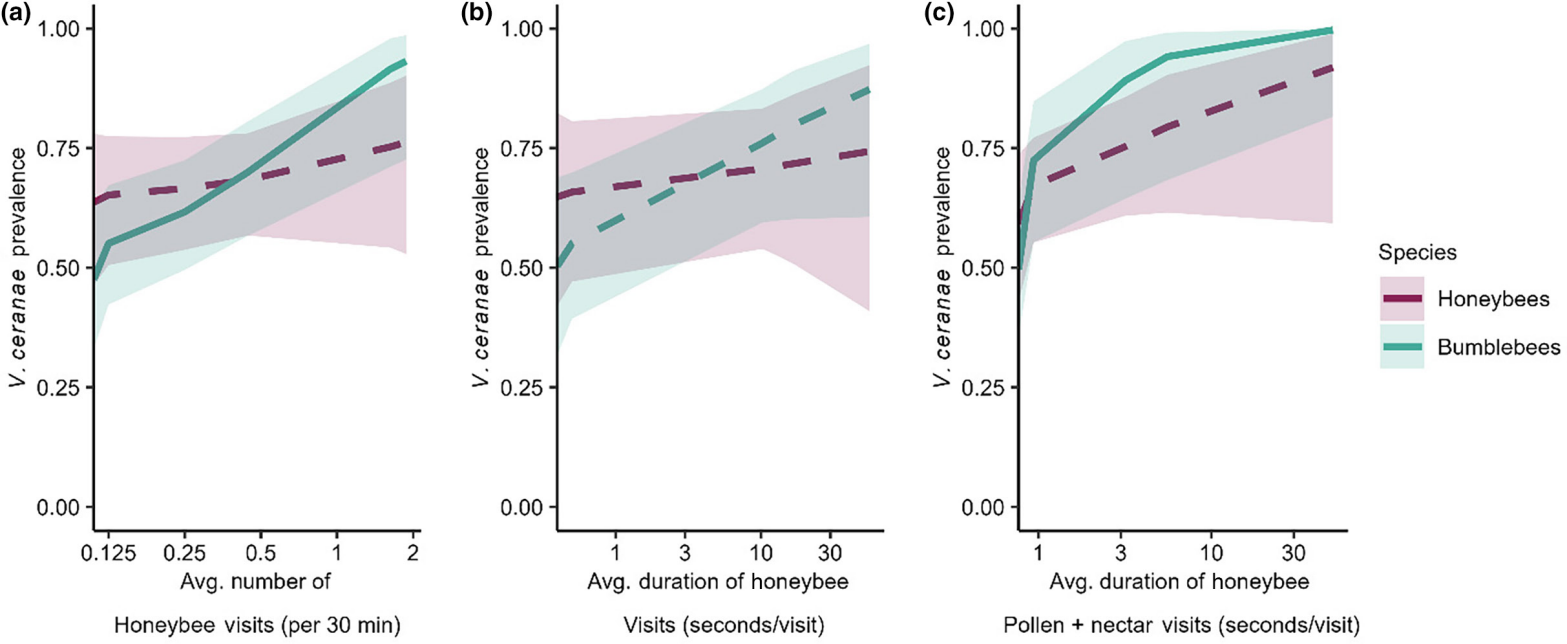
(a) The average number of honeybee visits to flowers was correlated with greater *Vairimorpha ceranae* prevalence in bumblebees (*p* = .006), but not in honeybees (*p* = .41). (b) The average total duration of time honeybees spent on flowers per visit did not correlate with *V. ceranae* prevalence in honeybees (*p* = .67) or bumblebees (*p* = .06). (c) The average duration of time honeybees spent per visit interacting with pollen and nectar simultaneously correlated with greater *V. ceranae* prevalence in bumblebees (*p* = .0116), but not *V. ceranae* prevalence in honeybees (*p* = .09). The number and duration of visits by honeybees (x axes) were converted to their original values and put on a log scale with zero values on the y axis for figure clarity. Significant slopes are indicated by solid lines, while insignificant slopes are indicated by dotted lines. Honeybee visits (per 30 min) were evaluated based on a 0.05 alpha threshold, while the visit duration (seconds/visit) was evaluated according to the Bonferroni‐corrected alpha threshold of 0.0125.

**FIGURE 4:**
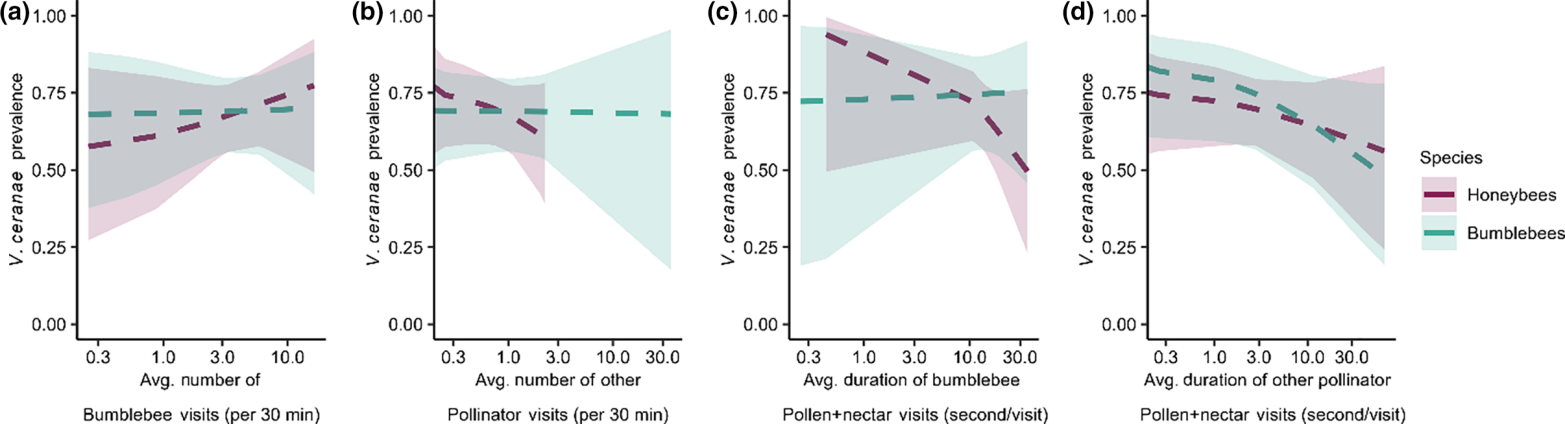
Neither the number of bumblebee and other pollinator visits per 30 min nor the duration per visit to pollen + nectar impacted *Vairimorpha ceranae* prevalence in honeybees or bumblebees. There was no change in *V. ceranae* prevalence in honeybees or bumblebees based on (a) average number of bumblebee visits (per 30 min), (b) average number of other pollinator visits (per 30 min), (c) average duration on pollen + nectar per bumblebee visit (second/visit), and (d) average duration on pollen + nectar per other pollinator visit (second/visit). X axes are on a log scale with the original values displayed, where zero values are on the y axis (if present). Significant slopes are indicated by solid lines, while insignificant slopes are indicated by dotted lines. In panel (b), the difference in the range of x axis values is caused by PR visit 1, where there were no honeybees detected (Table S5) but there were a very high number of other pollinator visits to squash flowers.

To determine whether floral visitation behaviors were linked with *V. ceranae* prevalence, we explored how the number of pollinator visits and the duration of time per visit spent interacting with certain parts of the flower correlated with *V. ceranae* prevalence in honeybees and bumblebees. Despite a lower number of honeybee visits compared to bumblebee visits (Figure 2a), the number of honeybee visits was the only factor that had a significant impact on *V. ceranae* prevalence. *Vairimorpha ceranae* prevalence in bumblebees was positively linked with honeybee flower visits (*p* = .006; Table 1, Figure 3a) but not bumblebee flower visits (*p* = .94, Figure 4a) or squash bee flower visits (*p* = .78, Appendix S1: Figure S3a). In contrast, *V. ceranae* prevalence in honeybees was not linked with honeybee flower visitation (*p* = .67, Table 1, Figure 3a), bumblebee flower visitation (*p* = .69, Figure 4a), or squash bee flower visitation (*p* = .23, Appendix S1: Figure S3a). *Vairimorpha ceranae* prevalence in honeybees and bumblebees was also not linked with flower visitation by other pollinator taxa (both *p* > .32, Figure 4b; Table 1, Tables S8 and S9).

**TABLE 1:** Scale standardized model estimates for the effects of the number of visits, total duration per visit, duration per visit of interactions with petals‐only, pollen‐only, and pollen and nectary simultaneously by honeybees, bumblebees, squash bees, and all other pollinators on *Vairimorpha ceranae* prevalence in honeybees and bumblebees.

Response variable	Visitation by	Visit number estimates	Total duration per visit estimates^a^	Petal duration per visit estimates^a^	Pollen duration per visit estimates^a^	Pollen & Nectary Duration per Visit Estimates^a^
*V. ceranae* prevalence in honeybees	Honeybees	0.257	0.167	0.038	−0.135	0.664
	Bumblebees	0.276	−0.195	0.556	−0.164	−0.716
	Squash bees	0.371	0.046	0.549	−0.576	−0.309
	Other	−0.252	0.044	−0.576	0.702	−0.301
*V. ceranae* prevalence in bumblebees	Honeybees	1.125**	0.700	0.598*	0.780*	1.802*
	Bumblebees	0.026	0.213	0.468	0.061	0.042
	Squash bees	−0.073	0.061	1.121*	−0.304	0.231
	Other	−0.008	−0.053	−0.599	0.170	−0.572

*Note*: Standardized estimates with a larger magnitude indicate a stronger relationship with *V. ceranae* prevalence within a given model. Full model output in Appendix S1: Tables S8 and S9. Significant estimates are bolded. Significant: **p < .01; trending: *p < .05.

^a^ Bonferroni‐corrected alpha threshold of 0.0125 for four comparisons applied to models with duration per visit main factors.

We also expected that greater amounts of time bees spent per visit on flowers and interacting with different aspects of the flower (e.g., petals, pollen, and pollen + nectar) would increase the *V. ceranae* prevalence by increasing the chances of parasite transmission. For the duration per visit models, we used a Bonferroni‐corrected significance threshold of 0.0125 because the durations per pollinator visit to each part of the flower were analyzed separately. *Vairimorpha ceranae* in honeybees and bumblebees was not associated with the total duration per visit of honeybees, bumblebees, squash bees, or other pollinators (Table 1, Figure 3b). We further explored this result by breaking down the total floral visit duration by the duration of time that bees spent interacting with different flower parts, including petals, pollen, and pollen + nectar to determine which specific behaviors contributed most to *V. ceranae* prevalence in each host species (Table 1; Appendix S1: Tables S8 and S9). *Vairimorpha ceranae* prevalence in bumblebees was higher the longer honeybees interacted with pollen + nectar (*p* = .0116; Figure 3c), despite no overall differences in time spent on pollen + nectar per visit among honeybees, bumblebees, and other pollinators (Figure 2d, Appendix S1: Figure S4, Table S4). *Vairimorpha ceranae* in both host species was not impacted by bumblebee, squash bee, or other pollinator duration spent per visit on pollen + nectar (Figure 4c,d; Table 1, Figure S3). *Vairimorpha ceranae* in honeybees and bumblebees was not correlated with the time per visit that bees spent on petals or pollen, regardless of bee species (Table 1, Figure S4).

## 4 DISCUSSION

We observed that honeybees, bumblebees, squash bees, and other pollinators differed in the number of visits to flowers and the duration per visit to petals and pollen but did not vary in the total length of time they spent on flowers. Honeybees had fewer flower visits than bumblebees, and all visiting pollinators spent similar amounts of time per visit interacting with the pollen and nectar simultaneously (Figure 2). Yet, the sites with more and longer honeybee visits to shared flowers had higher *V. ceranae* prevalence in bumblebees. Therefore, honeybee visitation to flowers appears to have a disproportionate impact on *V. ceranae* prevalence in local bumblebee populations. Visitation by bumblebees, squash bees, or other pollinators, in terms of the number of visits or time spent on flowers, was not associated with *V. ceranae* prevalence in either host species. These findings suggest honeybees may play an important role in the spread of *V. ceranae* to bumblebees through indirect contact via shared flowers in the natural environment. Such pathogen spillover from honeybees to bumblebees is likely to have negative consequences for bumblebee populations (Colla et al., 2006; Furst et al., 2014).

The spillover from honeybees to bumblebees may occur differently compared to transmission among honeybees. *Vairimorpha ceranae* is easily transmitted within honeybee hives when bees clean up fecal material, eat contaminated food, or perform trophallaxis (Chen et al., 2008; Higes et al., 2010). Further, drifting of honeybees among hives is known to occur and is thought to play a role in the transmission of parasites, including *V. ceranae* (Eberl & Muhammad, 2022; Higes et al., 2010). As *V. ceranae* is a well‐established concern for managed honeybee populations (Higes et al., 2013) and is thought to spill over from managed honeybee populations to wild bumblebee populations (Alger et al., 2018; Furst et al., 2014; Goulson & Hughes, 2015), high *V. ceranae* prevalence in honeybees may be driven by intraspecific transmission occurring among and within honeybee hives. In fact, the intensification of honeybee apiaries has a minimal impact on increasing *V. ceranae* prevalence among honeybees because prevalence is already quite high, estimated at about 90%, even for sites with low apiary intensity (Bartlett et al., 2019). In contrast, *V. ceranae* prevalence in bumblebees may be driven in part by parasite spillover from shared flowers with honeybees. Thus, spillover from honeybees to bumblebees could explain why honeybee visitation behavior was strongly correlated with *V. ceranae* prevalence in bumblebees, but not with prevalence in honeybees. However, especially considering that *V. ceranae* prevalence was quite high across both sites and species, further study in a more controlled manner is needed to learn more about how intraspecific transmission in honeybees and spillover to bumblebees may influence *V. ceranae* prevalence in the field, and whether there are other factors that influence the high prevalence. Future experiments could include studying *V. ceranae* in additional wild pollinator species that may differ in their competence for *V. ceranae* and a greater variety of sites, including sites with other crops or non‐agricultural sites, to better discern the reasons for the consistently high parasite prevalence observed in this study.

Our results are consistent with prior small‐scale lab experiments which demonstrated that pollinator parasites, including *V. ceranae*, are transmitted via contact with flowers (Durrer & Schmid‐Hempel, 1994; Graystock et al., 2015; Purkiss & Lach, 2019). Several recent studies have further shown that pollinator parasites are commonly found on flowers in the field, but their abundance varies based on flower morphology, the environment, and pollinator visitation patterns (Alger et al., 2019; Figueroa et al., 2019; Graystock et al., 2020; Russell et al., 2019). Furthermore, Graystock et al. (2015) experimentally showed that 23% of uninfected bumblebees that foraged on flowers recently visited by infected honeybees became infected with *V. ceranae*. This suggests that flowers can become hotspots for parasite dispersal once contaminated (Graystock et al., 2015). However, few studies have examined how differences in the pollinator community's floral visitation behaviors may impact parasite prevalence across multiple host species in nature (but see Graystock et al., 2020), and *V. ceranae*, in particular, has been neglected. As pathogens and parasites are key drivers of pollinator population decline (Potts et al., 2010), it is crucial to understand patterns of their transmission within and among pollinator species in the natural environment. Our findings corroborate prior experimental work and add that honeybee visitation to shared flowers—especially in areas with generally high *V. ceranae* prevalence in honeybees—facilitates greater *V. ceranae* spillover from managed honeybees to wild bumblebees in the natural environment.

While *V. ceranae* spillover via contaminated flowers seems likely, little is known about how pollinator interactions with different parts of inflorescences may affect the likelihood of *V. ceranae* transmission. We examined the association between *V. ceranae* prevalence and the duration per visit by honeybees, bumblebees, squash bees, and other pollinators to flower petals, nectaries, and pollen to explore which parts of inflorescences may have the greatest impact on *V. ceranae* spread. We found that higher *V. ceranae* prevalence in bumblebees was associated with longer durations of honeybee interactions per visit spent simultaneously contacting both the pollen and nectar of inflorescences (pollen + nectar). These visits were characterized by active foraging behavior for nectar and/or pollen while deeply embedded within the corolla of the large squash flowers. On the contrary, similar floral interactions by squash bees did not alter *V. ceranae* prevalence, suggesting that squash bees may not be an effective vector of these parasites. Controlled studies assessing *V. ceranae* in squash bees will be valuable as we currently know little about whether pollinators other than honeybees and bumblebees vector *V. ceranae*. Additionally, since there was no difference among honeybees, bumblebees, squash bees, and other pollinators in time spent per visit on the pollen + nectar (Figure 2d), our results suggest that time spent by honeybees on flowers disproportionately increases the likelihood of parasite spillover to bumblebees relative to time spent on flowers by bumblebees or other pollinators.

The length of time that infected honeybees spend closely interacting with both pollen and nectar—food resources that are consumed by many pollinator species—likely contributes to *V. ceranae* spore deposition on flower surfaces, which may be picked up and consumed by subsequent floral visitors. *Vairimorpha ceranae* is a fecal‐orally transmitted parasite (Chen et al., 2008; Smith, 2012) and bees commonly defecate on floral surfaces while foraging, with longer visits increasing the likelihood of defecation (Bodden et al., 2019). *Vairimorpha ceranae* has been detected in honeybee salivary glands (Chen et al., 2009) and viable and infectious *V. ceranae* spores have been found in the corbicular pollen of honeybees (Higes, Martín‐Hernández, Garrido‐Bailón, et al., 2008), suggesting that pollen can become contaminated during pollen collection. Therefore, it is possible that the pollen on the stamen may be a key hot spot for the deposition of *V. ceranae* by infected bees and the acquisition of this contaminated pollen by susceptible bees. However, this may not be the case for all susceptible bees, as squash bee visitation did not affect *V. ceranae* prevalence in honeybees or bumblebees, despite the fact that a species may act as a vector of the parasite between flowers even if it does not get infected itself (Davis et al., 2021). In contrast, nectar may be a poor location for pathogen transmission because high sugar concentrations can inhibit microbial growth and pathogen survival (Adler et al., 2021). We observed that honeybees and bumblebees seemed to spend more time on pollen + nectar interactions compared to pollen‐only or nectar‐only interactions (Appendix S1: Table S4), likely owing to their large size making it difficult to only contact one food source at a time. Therefore, the long visits with high floral contact during which honeybees and bumblebees foraged for pollen and nectar may have increased the chances for transmission to occur.

We did not observe any relationships between *V. ceranae* prevalence and the length of time pollinators spent interacting with only the petals or pollen. Though many bees spent time on the petals, bees were typically observed either resting or crawling on the petals for very short periods of time. Though other pollinator parasites are transmitted via floral petals (Figueroa et al., 2019), in our study petal‐only interactions were not linked with *V. ceranae* prevalence. Pathogenic spores can often survive well on floral surfaces (McArt et al., 2014), but their survival likely varies among different plant species, flower parts, and the centrality of the plant in the plant–pollinator network (Adler et al., 2018; Figueroa et al., 2019; Naughton et al., 2017; Palmer‐Young et al., 2016; Piot et al., 2020). Since this study only considered a single plant species (Cucurbit squash) and did not consider the plant–pollinator network, future studies are needed to empirically test how floral traits among different plant species affect pollinator–flower interactions, explore the distribution of spores on different floral surfaces in the natural environment, and determine the consequences for *V. ceranae* parasite dispersal via different parts of the inflorescences. In particular, additional plant species should be studied in order to understand how longer‐lasting flowers and flowers without specialized pollinators may influence the system.

In contrast to *V. ceranae* prevalence in bumblebees, we consistently found that *V. ceranae* prevalence in honeybees was not correlated with flower visitation by any species*. Vairimorpha ceranae* prevalence in honeybees was high at all sites (57.1%–81.3%; Appendix S1: Figure S2, Table S10), indicating that honeybees experience consistently high *V. ceranae* prevalence across the landscape. The spillover of *V. ceranae* from managed honeybee hosts to wild bumblebee populations would suggest that honeybees are a highly competent host for *V. ceranae* that could be facilitating transmission to other wild bee species in pollinator communities through indirect interactions on shared flowers. To gain a better understanding of this potential mechanism, further research should include more localized and controlled experiments on floral transmission, for example by capturing the individuals that visited a particular flower and subsequently testing both the bee and the flower for *V. ceranae*.

## 5 CONCLUSIONS

We found that *V. ceranae* prevalence in bumblebees was strongly associated with the floral visitation behaviors of honeybees. More honeybee visits and time spent interacting with both the pollen and nectar contributed to higher *V. ceranae* prevalence in bumblebees, despite honeybees visiting flowers less than bumblebees. These results suggest that even a few visits by honeybees to shared crop flowers may be having a disproportionately large effect on *V. ceranae* spillover from managed honeybee populations to wild bumblebee populations in agricultural landscapes. Our study provides a first look at how specific pollinator visitation behaviors on flowers impact the likelihood of parasite spillover among wild pollinators in nature. Understanding how the risk of *V. ceranae* exposure and potential infection for different bee species changes with regard to their shared floral landscape with honeybees is critical for reducing parasite spillover into declining wild bee populations. This knowledge may be particularly important in agricultural settings where managed honeybees and wild pollinators from the surrounding environment may frequently interact on crop flowers and nearby hedgerows, creating potential hotspots for parasite transmission on flowers.

## AUTHOR CONTRIBUTIONS

**Maryellen Zbrozek:** Conceptualization (equal); data curation (equal); formal analysis (equal); funding acquisition (supporting); investigation (equal); methodology (equal); project administration (equal); software (equal); validation (supporting); visualization (equal); writing – original draft (equal); writing – review and editing (equal). **Michelle L. Fearon:** Conceptualization (equal); data curation (equal); formal analysis (equal); funding acquisition (lead); investigation (equal); methodology (equal); project administration (equal); resources (equal); software (equal); supervision (equal); validation (lead); visualization (equal); writing – original draft (equal); writing – review and editing (equal). **Chloe Weise:** Investigation (supporting); methodology (supporting); validation (supporting); writing – review and editing (supporting). **Elizabeth Tibbetts:** Formal analysis (supporting); funding acquisition (supporting); resources (equal); supervision (equal); writing – review and editing (equal).

## CONFLICT OF INTEREST STATEMENT

The authors declare that they have no conflict of interest.

## Supporting information


Data S1.

## Data Availability

All data used for the analyses and figures in this manuscript are available on Dryad at the following DOI URL: https://doi.org/10.5061/dryad.vt4b8gtxt. All code used in calculations and analyses will be available at Zenodo: https://zenodo.org/record/7999767. All videos associated with the data in this manuscript will be available upon request from M.L. Fearon (mlfearon@umich.edu). All sequences generated in this study are available on GenBank Accession numbers OQ545564–OQ545565 and OQ550096–OQ550100.
