# Supplementary material for: Honeybee visitation to shared flowers increases Vairimorpha ceranae prevalence in bumblebees
Source: Ecol Evol. 2023 Sep 20;13(9):e10528. doi: 10.1002/ece3.10528 (PMC10511299; doi:10.1002/ece3.10528)
Supplement: Supplementary file 1 — Data S1. [file ECE3-13-e10528-s001.zip › ECE3_10528_APPENDIX S1.docx]

**APPENDIX S1**

**Table S1.** Field site abbreviation, farm name, dates of each visit to the field site (mm/dd/yyyy), zone, and Easting and Northing coordinates in the UTM GPS system. All field sites are located in the southeastern region of the Lower Peninsula of Michigan, USA. Permission from landowners was granted for all pollinator collection.

| Site Code | Farm Name | First Visit | Second visit | Zone | Easting | Northing |
| --- | --- | --- | --- | --- | --- | --- |
| BP | Brimley’s Pumpkin Patch | 8/10/2016 | 8/26/2016 | 16T | 714474 | 4716740 |
| K | Kapnick Orchards | 8/21/2016 | 8/28/2016 | 17T | 257729 | 4648607 |
| PR | Peacock Road Farms | 7/26/2016 | 8/23/2016 | 16T | 714244 | 4746884 |
| GT | Green Things Farm | 8/17/2016 | 8/24/2016 | 17T | 276741 | 4689607 |
| E | Erwin Orchards | 7/27/2016 | 8/22/2016 | 17T | 280997 | 4708908 |
| PL | Plymouth Orchards | 8/11/2016 | 8/30/2016 | 17T | 289557 | 4690343 |

**Table S2.** The sampling of different *Bombus* spp. across the two visits to each site. *Bombus* *impatiens* was the most common and abundant *Bombus* spp., and all other *Bombus* spp. were rare and sporadically found across most of the sites.

| Site | Visit | *B. auricomus* | *B. bimaculatus* | *B. fervidus* | *B. griseocollis* | *B. impatiens* | *B. pensylvanicus* | *B. sandersoni* | *B. vagans* |
| --- | --- | --- | --- | --- | --- | --- | --- | --- | --- |
| BP | **1** | 0 | 0 | 0 | 1 | 37 | 0 | 0 | 0 |
| BP | **2** | 0 | 0 | 0 | 1 | 89 | 0 | 2 | 0 |
| E | **1** | 0 | 1 | 1 | 0 | 4 | 1 | 0 | 0 |
| E | **2** | 3 | 0 | 0 | 1 | 72 | 0 | 0 | 0 |
| GT | **1** | 0 | 0 | 0 | 1 | 39 | 0 | 0 | 0 |
| GT | **2** | 0 | 0 | 1 | 0 | 66 | 0 | 0 | 1 |
| K | **1** | 0 | 0 | 0 | 0 | 53 | 0 | 0 | 0 |
| K | **2** | 0 | 0 | 0 | 0 | 36 | 0 | 0 | 0 |
| PL | **1** | 0 | 0 | 0 | 0 | 18 | 0 | 0 | 0 |
| PL | **2** | 0 | 0 | 0 | 2 | 87 | 0 | 0 | 0 |
| PR | **1** | 0 | 0 | 0 | 0 | 3 | 0 | 0 | 0 |
| PR | **2** | 0 | 0 | 0 | 0 | 164 | 0 | 0 | 0 |

**Table S3.** Morphospecies classifications used to identify individuals visiting squash flowers in the visitation videos. For analyses, visitations by all other pollinator groups excluding *Apis mellifera*, *Bombus* spp., and *Eucera pruinosa*, were combined into the ‘Other’ category.

| **Code** | **Possible included species** | **Total Num. Visits** | **Characteristics** |
| --- | --- | --- | --- |
| APIS | *Apis mellifera* | 50 |  |
| AUGO | *Augochlora*, *Augochlorella*, *Augochloropsis* | 232 | Small green halictid |
| BOMB | *Bombus* | 477 |  |
| HALI | *Halictus*, *Lasioglossum* | 128 | Small non-green halictid |
| HFLY | Hover fly | 13 |  |
| MELI | *Melissodes* | 2 |  |
| PEPO | *Eucera (=Peponapis) pruinosa* | 180 | Specialist squash bee |
| TRIE | *Triepeolus* | 1 | Cuckoo bee, parasitizes *Eucera* spp. |
| VESP | *Vespula* (wasp) | 1 |  |
|  |  |  |  |

**Table S4.** Means and ranges of each visitation variable for honeybees, bumblebees, squash bees, and all other pollinator taxa among flowers at each site. Durations per visit are calculated as the mean number of seconds that a bee species visited the flower per visit (i.e., average number of seconds honeybees spent doing a given behavior divided by the number of honeybee visits to the flower).

|  | **SITE** | **Honeybees** | **Bumblebees** | **Squash bees** | **Other Pollinators** |
| --- | --- | --- | --- | --- | --- |
| **VISIT NUMBER PER 30 MIN** | **BP** | 1.75 (0, 7) | 9.25 (0, 25) | 0.69 (0, 4) | 1.06 (0, 4) |
|  | **E** | 0 (0,0) | 1.63 (0, 8) | 6.94 (0, 43) | 0.38 (0, 3) |
|  | **GT** | 0.24 (0, 3) | 4.47 (0, 15) | 0.24 (0, 2) | 1.35 (0, 8) |
|  | **K** | 0 (0,0) | 1.31 (0, 6) | 0.06 (0, 1) | 1.25 (0, 4) |
|  | **PL** | 1 (0, 7) | 4.44 (0, 15) | 2.13 (0, 12) | 1.31 (0, 5) |
|  | **PR** | 0.13(0, 2) | 8.44 (0, 38) | 1.19 (0, 12) | 18.13 (0, 135) |
| **TOTAL DURATION PER VISIT** | **BP** | 34.33 (0, 219.3) | 19.57 (0, 70) | 10.79 (0, 126.5) | 30.37 (0, 318) |
|  | **E** | 0 (0,0) | 20.39 (0, 222.8) | 21.89 (0, 166.5) | 4.79 (0, 54.7) |
|  | **GT** | 5.39 (0, 90.7) | 18.90 (0, 42.3) | 3.00 (0, 40) | 14.60 (0, 85) |
|  | **K** | 0 (0,0) | 15.41 (0, 65) | 0.31 (0, 5) | 32.62 (0, 103.3) |
|  | **PL** | 8.78 (0. 64.3) | 20.63 (0, 107) | 3.45 (0, 15) | 15.72 (0, 117.3) |
|  | **PR** | 5.28 (0, 84.5) | 11.99 (0, 40.3) | 10.35 (0, 137) | 65.47 (0, 3.9) |
| **PETAL-ONLY DURATION PER VISIT** | **BP** | 4.03 (0, 23) | 0.83 (0, 3.1) | 1.23 (0, 10.5) | 9. 43 (0, 50.5) |
|  | **E** | 0 (0,0) | 0.29 (0, 4) | 0.04 (0, 0.5) | 1.96 (0, 12) |
|  | **GT** | 5.10 (0, 86.7) | 0.98 (0, 6) | 0.12 (0, 1) | 6.96 (0, 43) |
|  | **K** | 0 (0,0) | 0.85 (0, 4.3) | 0.31 (0, 5) | 11.57 (0, 92.3) |
|  | **PL** | 2.31 (0, 11.7) | 1.60 (0, 4) | 0.67 (0, 6) | 9.27 (0, 58.5) |
|  | **PR** | 3.94 (0, 63) | 0.55 (0, 4) | 0.01 (0, 0.2) | 19.27 (0, 92.6) |
| **NECTAR-ONLY DURATION PER VISIT** | **BP** | 0.29 (0, 2.5) | 0.03 (0, 0.55) | 0.08 (0, 1.25) | 2.14 (0, 21.5) |
|  | **E** | 0 (0,0) | 0 (0,0) | 0 (0,0) | 0.25 (0, 4) |
|  | **GT** | 0 (0,0) | 0 (0,0) | 0 (0,0) | 5.29 (0, 46.4) |
|  | **K** | 0 (0,0) | 0.03 (0, 0.5) | 0 (0,0) | 18.34 (0, 80) |
|  | **PL** | 4.66 (0, 52.7) | 0.67 (0, 8.4) | 0 (0,0) | 6.23 (0, 99.8) |
|  | **PR** | 0.66 (0, 10.5) | 0 (0,0) | 0 (0,0) | 15.75 (0, 116) |
| **POLLEN-ONLY DURATION PER VISIT** | **BP** | 2.00 (0, 11) | 2.50 (0, 6.4) | 0.69 (0, 4) | 20.41 (0, 308.5) |
|  | **E** | 0 (0,0) | 1.30 (0, 7) | 7.15 (0, 76) | 15.27 (0, 152) |
|  | **GT** | 0.29 (0, 4) | 2.63 (0, 9) | 0.41 (0, 6) | 2.20 (0, 24) |
|  | **K** | 0 (0,0) | 0.80 (0, 3) | 0 (0, 0) | 2.67 (0, 14) |
|  | **PL** | 0.22 (0, 3) | 2.10 (0, 14.3) | 1.21 (0, 12.1) | 2.56 (0, 27.25) |
|  | **PR** | 0.22 (0, 3.5) | 1.26 (0, 4.6) | 4.41 (0, 66.8) | 9.09 (0, 26.3) |
| **POLLEN+NECTAR DURATION PER VISIT** | **BP** | 28.01 (0, 197.8) | 16.17 (0, 67) | 8.80 (0, 113.5) | 7.17 (0, 75.7) |
|  | **E** | 0 (0,0) | 17.84 (0, 218.8) | 14.70 (0, 90) | 33.54 (0, 228) |
|  | **GT** | 0 (0,0) | 15.29 (0, 39.7) | 2.47 (0, 34) | 1.34 (0, 14.8) |
|  | **K** | 0 (0,0) | 13.66 (0, 60.5) | 0 (0, 0) | 0.13 (0, 2) |
|  | **PL** | 1.59 (0, 17.4) | 14.20 (0, 103) | 1.57 (0, 9) | 0.92 (0, 7) |
|  | **PR** | 0.47 (0, 7.5) | 9.93 (0, 37.8) | 5.93 (0, 70.3) | 22.17 (0, 295) |

**Table S5.** Numbers of honeybee (*Apis mellifera*) and bumblebee (*Bombus* spp.) individuals tested and positive for *V. ceranae* presence across six sites and two visits per site. We aimed to sample eight individuals per species per visit to each site where possible (target N = 16 per species per site), but there was some variation in the abundance of honeybees and bumblebees across visits to each field site.

| Species; | | honeybees | | Bumblebees | | Combined | |
| --- | --- | --- | --- | --- | --- | --- | --- |
| Site | **VISIT** | **Tested** | **Positive** | **Tested** | **Positive** | **Tested** | **Positive** |
| BP | 1 | 8 | *4* | 8 | 7 | 16 | 11 |
| BP | 2 | 8 | *7* | 8 | 8 | 16 | 15 |
| E | 1 | 8 | *6* | 7* | 2 | 15 | 8 |
| E | 2 | 8 | *4* | 8 | 4 | 16 | 8 |
| GT | 1 | 7 | *3* | 8 | 4 | 15 | 7 |
| GT | 2 | 5 | *5* | 7 | 4 | 12 | 9 |
| K | 1 | 3 | *2* | 7 | 4 | 10 | 6 |
| K | 2 | 5 | *3* | 8 | 4 | 13 | 7 |
| PL | 1 | 8 | *5* | 7 | 5 | 15 | 10 |
| PL | 2 | 8 | *8* | 8 | 8 | 16 | 16 |
| PR | 1 | 0 | *0* | 2 | 1 | 2 | 1 |
| PR | 2 | 7 | *4* | 8 | 4 | 15 | 8 |
| *TOTAL* | | **75** | ***51*** | **86** | ***55*** | **161** | ***106*** |

* This site visit includes 3 individuals that were identified as *Bombus fervidus, Bombus bimaculatus,* and *Bombus pensylvanicus* species, rather than *Bombus impatiens*.

**Table S6.** Full model output for each zero-inflated hurdle GLMM for pollinator species (honeybees, bumblebees, squash bees, or other pollinators) effects on the number of visits per 30 min and total, petal, pollen, and pollen+nectar durations per visit. Honeybees are considered the reference in this model, and the estimates and p-values reported below are relative to honeybees (see Table S7 for the post-hoc analysis of the pairwise differences among pollinator species). The table includes the model estimate, standard error, z value, and p-value for each main effect in the conditional and zero-inflated portions of the models, as well as the variance and standard deviation for the nested random effects of each observed flower within site visit (i.e., first or second visit to each site) within site. All models used a negative binomial distribution with a log link function. Some models have singular random effects, indicating no variation in all or some of the nested random effects. Significant p-values are bolded.

| Response variable | Main Effect | Estimate | Std Error | z value | P-value | Random Effect | Variance | Std Dev. |
| --- | --- | --- | --- | --- | --- | --- | --- | --- |
| *Number of visits per 30 min* | **Conditional model:** |  |  |  |  |  |  |  |
|  | Intercept | 0.35 | 0.47 | 0.75 | 0.451 | FlowerID:Visit:Site | 2.00E-09 | 4.47E-05 |
|  | Bumblebees | 1.50 | 0.39 | 3.80 | **0.00014** | Visit:Site | 0.78 | 0.88 |
|  | Squash bees | 0.50 | 0.59 | 0.85 | 0.397 | Site | 2.67E-06 | 0.0016 |
|  | Other Pollinators | 0.14 | 0.48 | 0.30 | 0.764 |  |  |  |
|  | **Zero-inflation model:** |  |  |  |  |  |  |  |
|  | Intercept | 0.57 | 0.42 | 1.37 | 0.170 |  |  |  |
|  | Bumblebees | -2.14 | 0.57 | -3.77 | **0.00016** |  |  |  |
|  | Squash bees | -0.39 | 0.55 | -0.71 | 0.477 |  |  |  |
|  | Other Pollinators | -2.42 | 1.32 | -1.84 | 0.066 |  |  |  |
| *Total duration per visit* | **Conditional model:** |  |  |  |  |  |  |  |
|  | Intercept | 3.21 | 0.35 | 9.29 | **<0.0001** | FlowerID:Visit:Site | 0.02 | 0.13 |
|  | Bumblebees | -0.31 | 0.37 | -0.85 | 0.396 | Visit:Site | 0.09 | 0.30 |
|  | Squash bees | -0.37 | 0.44 | -0.85 | 0.397 | Site | 3.59E-07 | 0.0006 |
|  | Other Pollinators | 0.35 | 0.38 | 0.91 | 0.361 |  |  |  |
|  | **Zero-inflation model:** |  |  |  |  |  |  |  |
|  | Intercept | 1.24 | 0.27 | 4.53 | **<0.0001** |  |  |  |
|  | Bumblebees | -2.62 | 0.49 | -5.30 | **<0.0001** |  |  |  |
|  | Squash bees | -0.53 | 0.36 | -1.44 | 0.149 |  |  |  |
|  | Other Pollinators | -1.40 | 0.35 | -3.97 | **<0.0001** |  |  |  |
| *Duration on Petals per visit* | **Conditional model:** |  |  |  |  |  |  |  |
|  | Intercept | 1.49 | 0.51 | 2.94 | **0.003** | FlowerID:Visit:Site | 0.23 | 0.48 |
|  | Bumblebees | -1.96 | 0.44 | -4.42 | **<0.0001** | Visit:Site | 4.06E-08 | 0.0002 |
|  | Squash bees | -2.17 | 0.51 | -4.28 | **<0.0001** | Site | 0.32 | 0.57 |
|  | Other Pollinators | 0.75 | 0.45 | 1.65 | 0.099 |  |  |  |
|  | **Zero-inflation model:** |  |  |  |  |  |  |  |
|  | Intercept | 1.14 | 0.32 | 3.55 | **0.00038** |  |  |  |
|  | Bumblebees | -25.52 | 60820.4 | 0.00 | 0.9997 |  |  |  |
|  | Squash bees | -0.65 | 0.59 | -1.11 | 0.266 |  |  |  |
|  | Other Pollinators | -1.67 | 0.41 | -4.02 | **<0.0001** |  |  |  |
| *Duration on Pollen per visit* | **Conditional model:** |  |  |  |  |  |  |  |
|  | Intercept | -0.22 | 0.46 | -0.48 | 0.629 | FlowerID:Visit:Site | 0.30 | 0.55 |
|  | Bumblebees | 0.63 | 0.44 | 1.45 | 0.147 | Visit:Site | 2.35E-07 | 0.0005 |
|  | Squash bees | 1.13 | 0.55 | 2.06 | **0.039** | Site | 0.17 | 0.41 |
|  | Other Pollinators | 2.66 | 0.48 | 5.55 | **<0.0001** |  |  |  |
|  | **Zero-inflation model:** |  |  |  |  |  |  |  |
|  | Intercept | 0.78 | 0.48 | 1.63 | 0.104 |  |  |  |
|  | Bumblebees | -17.34 | 2047.78 | -0.01 | 0.993 |  |  |  |
|  | Squash bees | -0.19 | 0.57 | -0.34 | 0.736 |  |  |  |
|  | Other Pollinators | -0.70 | 0.53 | -1.33 | 0.184 |  |  |  |
| *Duration on Pollen + Nectar per visit* | **Conditional model:** |  |  |  |  |  |  |  |
|  | Intercept | 2.61 | 0.50 | 5.22 | **<0.0001** | FlowerID:Visit:Site | 0.70 | 0.84 |
|  | Bumblebees | 0.26 | 0.47 | 0.55 | 0.582 | Visit:Site | 0.37 | 0.61 |
|  | Squash bees | -0.64 | 0.50 | -1.28 | 0.200 | Site | 4.83E-08 | 0.0002 |
|  | Other Pollinators | 0.21 | 0.51 | 0.40 | 0.687 |  |  |  |
|  | **Zero-inflation model:** |  |  |  |  |  |  |  |
|  | Intercept | 1.92 | 0.33 | 5.78 | **<0.0001** |  |  |  |
|  | Bumblebees | -2.45 | 0.41 | -6.04 | **<0.0001** |  |  |  |
|  | Squash bees | -0.97 | 0.43 | -2.28 | **0.023** |  |  |  |
|  | Other Pollinators | -1.19 | 0.40 | -2.96 | **0.003** |  |  |  |

**Table S7.** Pairwise contrasts between each pollinator species group (honeybees, bumblebees, squash bees, and other pollinators) for each pollinator visitation behavior metric, including number of visits per 30 min, total duration per visit, and duration per visit on petals, pollen, and pollen+nectar. Odds ratios are shown for the pairwise difference between the two host species compared in each row and are calculated on the log scale. P values are Tukey adjusted for comparing a family of three, and significant p-values are bolded. Data for these tests are shown in Figure 2 and Figure S1.

| **Response variable** | **Contrast** | **Ratio** | **Std Error** | **DF** | **z value** | **P-value** |
| --- | --- | --- | --- | --- | --- | --- |
| ***Number of visits per 30 min*** | Honeybees / Bumblebees | 0.224 | 0.088 | Inf | -3.803 | **0.0008** |
|  | Honeybees / Squash bees | 0.605 | 0.359 | Inf | -0.848 | 0.832 |
|  | Honeybees / Other Pollinators | 0.867 | 0.414 | Inf | -0.300 | 0.991 |
|  | Bumblebees / Squash bees | 2.699 | 1.334 | Inf | 2.010 | 0.184 |
|  | Bumblebees / Other Pollinators | 3.869 | 1.439 | Inf | 3.637 | **0.0016** |
|  | Squash bees / Other Pollinators | 1.433 | 0.668 | Inf | 0.773 | 0.867 |
| ***Total duration per visit*** | Honeybees / Bumblebees | 1.366 | 0.502 | Inf | 0.849 | 0.831 |
|  | Honeybees / Squash bees | 1.449 | 0.634 | Inf | 0.847 | 0.832 |
|  | Honeybees / Other Pollinators | 0.704 | 0.271 | Inf | -0.914 | 0.798 |
|  | Bumblebees / Squash bees | 1.061 | 0.380 | Inf | 0.164 | 0.998 |
|  | Bumblebees / Other Pollinators | 0.515 | 0.141 | Inf | -2.427 | 0.072 |
|  | Squash bees / Other Pollinators | 0.486 | 0.174 | Inf | -2.017 | 0.182 |
| ***Duration on Petals per visit*** | Honeybees / Bumblebees | 7.121 | 3.163 | Inf | 4.419 | **0.0001** |
|  | Honeybees / Squash bees | 8.747 | 4.437 | Inf | 4.275 | **0.0001** |
|  | Honeybees / Other Pollinators | 0.472 | 0.215 | Inf | -1.652 | 0.3497 |
|  | Bumblebees / Squash bees | 1.228 | 0.560 | Inf | 0.451 | 0.9694 |
|  | Bumblebees / Other Pollinators | 0.066 | 0.017 | Inf | -10.824 | **<0.0001** |
|  | Squash bees / Other Pollinators | 0.054 | 0.025 | Inf | -6.346 | **<0.0001** |
| ***Duration on Pollen per visit*** | Honeybees / Bumblebees | 0.531 | 0.232 | Inf | -1.452 | 0.467 |
|  | Honeybees / Squash bees | 0.323 | 0.177 | Inf | -2.062 | 0.166 |
|  | Honeybees / Other Pollinators | 0.070 | 0.034 | Inf | -5.548 | **<0.0001** |
|  | Bumblebees / Squash bees | 0.608 | 0.248 | Inf | -1.218 | 0.615 |
|  | Bumblebees / Other Pollinators | 0.132 | 0.037 | Inf | -7.175 | **<0.0001** |
|  | Squash bees / Other Pollinators | 0.217 | 0.084 | Inf | -3.946 | **0.0005** |
| ***Duration on Pollen + Nectar per visit*** | Honeybees / Bumblebees | 0.773 | 0.361 | Inf | -0.551 | 0.946 |
|  | Honeybees / Squash bees | 1.896 | 0.945 | Inf | 1.283 | 0.574 |
|  | Honeybees / Other Pollinators | 0.813 | 0.417 | Inf | -0.403 | 0.978 |
|  | Bumblebees / Squash bees | 2.452 | 1.090 | Inf | 2.018 | 0.181 |
|  | Bumblebees / Other Pollinators | 1.051 | 0.466 | Inf | 0.113 | 0.9995 |
|  | Squash bees / Other Pollinators | 0.429 | 0.156 | Inf | -2.322 | 0.093 |

**Table S8.** Full model output for each GLMM for *V. ceranae* prevalence in honeybees, including the model estimate, standard error, z value, and p-value for each main effect in the models, as well as the variance and standard deviation for the nested random effects of each site visit (i.e., first or second visit to each site) within site. There are separate models for each pollinator behavior variable. Some models have singular random effects, indicating no variation in all or some of the nested random effects. Significant p-values are bolded, and all duration per visit models use the Bonferroni-corrected alpha threshold of 0.0125 instead of 0.05. Note that data on the nectar-only interactions are only included in the Appendix and were not part of the analyses in the main text and are not included as a group in the Bonferroni correction for four comparisons (Table 1).

| Response variable | Main Effect | Estimate | Std Error | z value | P-value | Random Effect | Variance | Std Dev. |
| --- | --- | --- | --- | --- | --- | --- | --- | --- |
| *V. ceranae* in honeybees | Intercept | 0.788 | 0.256 | 3.077 | **0.002** | Site:Visit | 0 | 0 |
|  | Number of honeybee visits per 30 min | 0.257 | 0.309 | 0.832 | 0.406 | Site | 0 | 0 |
|  | Number of bumblebee visits per 30 min | 0.276 | 0.356 | 0.774 | 0.439 |  |  |  |
|  | Number of squash bee visits per 30 min | 0.371 | 0.307 | 1.209 | 0.227 |  |  |  |
|  | Number of other visits per 30 min | -0.252 | 0.256 | -0.987 | 0.323 |  |  |  |
| *V. ceranae* in honeybees | Intercept | 0.780 | 0.274 | 2.851 | **0.004** | Site:Visit | 0.062 | 0.249 |
|  | Total duration per visit of honeybee visits | 0.167 | 0.393 | 0.425 | 0.671 | Site | 1.90E-09 | 4.35E-05 |
|  | Total duration per visit of bumblebee visits | -0.195 | 0.482 | -0.404 | 0.686 |  |  |  |
|  | Total duration per visit of squash bee visits | 0.046 | 0.462 | 0.099 | 0.921 |  |  |  |
|  | Total duration per visit of other visits | 0.044 | 0.308 | 0.141 | 0.888 |  |  |  |
| *V. ceranae* in honeybees | Intercept | 0.864 | 0.276 | 3.131 | **0.002** | Site:Visit | 0 | 0 |
|  | Duration per visit of honeybee petal-only interactions | 0.038 | 0.280 | 0.136 | 0.892 | Site | 0 | 0 |
|  | Duration per visit of bumblebee petal-only interactions | 0.556 | 0.403 | 1.379 | 0.168 |  |  |  |
|  | Duration per visit of squash bee petal-only interactions | 0.549 | 0.355 | 1.545 | 0.122 |  |  |  |
|  | Duration per visit of other petal-only interactions | -0.576 | 0.384 | -1.500 | 0.134 |  |  |  |
| *V. ceranae* in honeybees | Intercept | 1.324 | 1.137 | 1.164 | 0.244 | Site:Visit | 0 | 0 |
|  | Duration per visit of honeybee nectar-only interactions | 0.048 | 0.779 | 0.061 | 0.951 | Site | 0 | 0 |
|  | Duration per visit of bumblebee nectar-only interactions | 2.105 | 3.058 | 0.688 | 0.491 |  |  |  |
|  | Duration per visit of squash bee nectar-only interactions | 0.269 | 0.429 | 0.627 | 0.531 |  |  |  |
|  | Duration per visit of other nectar-only interactions | -0.245 | 0.318 | -0.768 | 0.442 |  |  |  |
| *V. ceranae* in honeybees | Intercept | 0.800 | 0.259 | 3.090 | **0.002** | Site:Visit | 0.139 | 0.372 |
|  | Duration per visit of honeybee pollen-only interactions | -0.135 | 0.286 | -0.471 | 0.637 | Site | 0 | 0 |
|  | Duration per visit of bumblebee pollen-only interactions | -0.164 | 0.364 | -0.451 | 0.652 |  |  |  |
|  | Duration per visit of squash bee pollen-only interactions | -0.576 | 0.459 | -1.256 | 0.209 |  |  |  |
|  | Duration per visit of other pollen-only interactions | 0.702 | 0.399 | 1.761 | 0.078 |  |  |  |
| *V. ceranae* in honeybees | Intercept | 0.806 | 0.260 | 3.095 | **0.002** | Site:Visit | 0 | 0 |
|  | Duration per visit of honeybee pollen+nectar interactions | 0.664 | 0.397 | 1.670 | 0.095 | Site | 0 | 0 |
|  | Duration per visit of bumblebee pollen+nectar interactions | -0.716 | 0.502 | -1.428 | 0.153 |  |  |  |
|  | Duration per visit of squash bee pollen+nectar interactions | -0.309 | 0.577 | -0.535 | 0.592 |  |  |  |
|  | Duration per visit of other pollen+nectar interactions | -0.301 | 0.364 | -0.828 | 0.408 |  |  |  |

**Table S9.** Full model output for each GLMM for *V. ceranae* prevalence in *Bombus* spp*.* (bumblebees), including the model estimate, standard error, z value, and p-value for each main effect in the models, as well as the variance and standard deviation for the nested random effects of each site visit within site. Some models have singular random effects, indicating no variation in all or some of the nested random effects. Significant p-values are bolded, and all duration per visit models use the Bonferroni-corrected alpha threshold of 0.0125 instead of 0.05. Note that data on the nectar-only interactions are only included in the Appendix and were not part of the analyses in the main text and are not included as a group in the Bonferroni correction for four comparisons (Table 1).

| Response variable | Main Effect | Estimate | Std Error | z value | P-value | Random Effect | Variance | Std Dev. |
| --- | --- | --- | --- | --- | --- | --- | --- | --- |
| *V. ceranae* in bumblebees | Intercept | 0.803 | 0.287 | 2.799 | **0.005** | Site:Visit | 2.57E-15 | 5.07E-08 |
|  | Number of honeybee visits per 30 min | 1.125 | 0.409 | 2.754 | **0.006** | Site | 0 | 0 |
|  | Number of bumblebee visits per 30 min | 0.026 | 0.329 | 0.079 | 0.937 |  |  |  |
|  | Number of squash bee visits per 30 min | -0.073 | 0.256 | -0.286 | 0.775 |  |  |  |
|  | Number of other visits per 30 min | -0.008 | 0.235 | -0.036 | 0.972 |  |  |  |
| *V. ceranae* in bumblebees | Intercept | 0.663 | 0.253 | 2.617 | **0.009** | Site:Visit | 0 | 0 |
|  | Total duration per visit of honeybee visits | 0.700 | 0.377 | 1.856 | 0.063 | Site | 0.017 | 0.129 |
|  | Total duration per visit of bumblebee visits | 0.213 | 0.454 | 0.468 | 0.639 |  |  |  |
|  | Total duration per visit of squash bee visits | 0.061 | 0.437 | 0.139 | 0.890 |  |  |  |
|  | Total duration per visit of other visits | -0.053 | 0.290 | -0.184 | 0.854 |  |  |  |
| *V. ceranae* in bumblebees | Intercept | 0.867 | 0.310 | 2.793 | **0.005** | Site:Visit | 0 | 0 |
|  | Duration per visit of honeybee petal-only interactions | 0.598 | 0.275 | 2.176 | 0.030 | Site | 0 | 0 |
|  | Duration per visit of bumblebee petal-only interactions | 0.468 | 0.352 | 1.327 | 0.184 |  |  |  |
|  | Duration per visit of squash bee petal-only interactions | 1.121 | 0.521 | 2.151 | 0.031 |  |  |  |
|  | Duration per visit of other petal-only interactions | -0.599 | 0.355 | -1.684 | 0.092 |  |  |  |
| *V. ceranae* in bumblebees | Intercept | 2.518 | 115.86 | 0.022 | 0.983 | Site:Visit | 0 | 0 |
|  | Duration per visit of honeybee nectar-only interactions | 0.373 | 0.626 | 0.596 | 0.551 | Site | 0 | 0 |
|  | Duration per visit of bumblebee nectar-only interactions | 1.214 | 1.488 | 0.816 | 0.415 |  |  |  |
|  | Duration per visit of squash bee nectar-only interactions | 5.516 | 363.90 | 0.015 | 0.988 |  |  |  |
|  | Duration per visit of other nectar-only interactions | -0.135 | 0.253 | -0.536 | 0.592 |  |  |  |
| *V. ceranae* in bumblebees | Intercept | 0.711 | 0.293 | 2.425 | 0.015 | Site:Visit | 0 | 0 |
|  | Duration per visit of honeybee pollen-only interactions | 0.780 | 0.367 | 2.128 | 0.033 | Site | 0.097 | 0.312 |
|  | Duration per visit of bumblebee pollen-only interactions | 0.061 | 0.352 | 0.174 | 0.862 |  |  |  |
|  | Duration per visit of squash bee pollen-only interactions | -0.304 | 0.424 | -0.716 | 0.474 |  |  |  |
|  | Duration per visit of other pollen-only interactions | 0.170 | 0.452 | 0.377 | 0.706 |  |  |  |
| *V. ceranae* in bumblebees | Intercept | 1.077 | 0.416 | 2.592 | **0.010** | Site:Visit | 3.79E-17 | 6.16E-09 |
|  | Duration per visit of honeybee pollen+nectar interactions | 1.802 | 0.714 | 2.524 | **0.0116** | Site | 0 | 0 |
|  | Duration per visit of bumblebee pollen+nectar interactions | 0.042 | 0.413 | 0.102 | 0.919 |  |  |  |
|  | Duration per visit of squash bee pollen+nectar interactions | 0.231 | 0.541 | 0.426 | 0.670 |  |  |  |
|  | Duration per visit of other pollen+nectar interactions | -0.572 | 0.343 | -1.666 | 0.096 |  |  |  |

**Table S10.** The *V. ceranae* prevalence (%) for honeybees and bumblebees at each site, including the lower and upper asymptotic confidence intervals, and the number of *V. ceranae* positive samples and the number of samples tested per host species.

| Genus | Site | Estimated Prevalence | Lower CL | Upper CL | No. *V. ceranae* Positive | No. Samples Tested |
| --- | --- | --- | --- | --- | --- | --- |
| Honeybees | BP | 68.8 | 43.4 | 86.8 | 11 | 16 |
| Honeybees | E | 62.5 | 36.8 | 82.5 | 10 | 16 |
| Honeybees | GT | 66.7 | 36.6 | 87.7 | 8 | 12 |
| Honeybees | K | 62.5 | 28.9 | 88.9 | 5 | 8 |
| Honeybees | PL | 81.3 | 56.6 | 94.7 | 13 | 16 |
| Honeybees | PR | 57.1 | 22.5 | 87.1 | 4 | 7 |
| Bumblebees | BP | 93.8 | 70.0 | 99.7 | 15 | 16 |
| Bumblebees | E | 40.0 | 18.6 | 66.8 | 6 | 15 |
| Bumblebees | GT | 53.3 | 29.0 | 78.5 | 8 | 15 |
| Bumblebees | K | 53.3 | 29.0 | 78.5 | 8 | 15 |
| Bumblebees | PL | 86.7 | 60.6 | 97.6 | 13 | 15 |
| Bumblebees | PR | 50.0 | 22.2 | 77.8 | 5 | 10 |

**
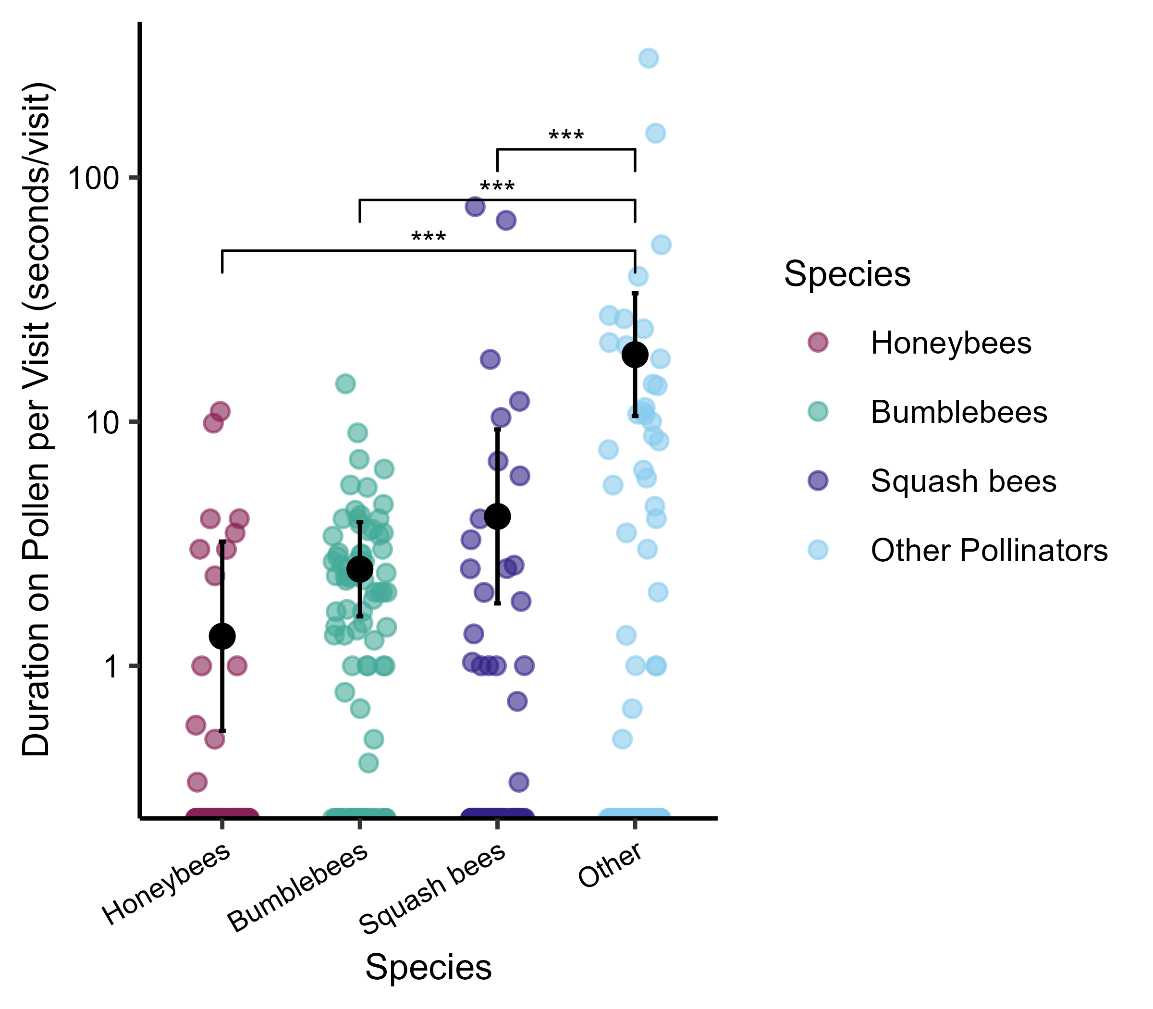
**

**Figure S1.** Other pollinators spent more time per visit (seconds/visit) on pollen than honeybees, bumblebees, and squash bees. Significant differences are indicated by the number of stars for each pair (Appendix S1: Table S7). The y-axis is log scaled, and zero values are shown along the x-axis. Colored points are the raw data per flower observed, and the black points are the model predicted marginal means with 95% confidence intervals.

**
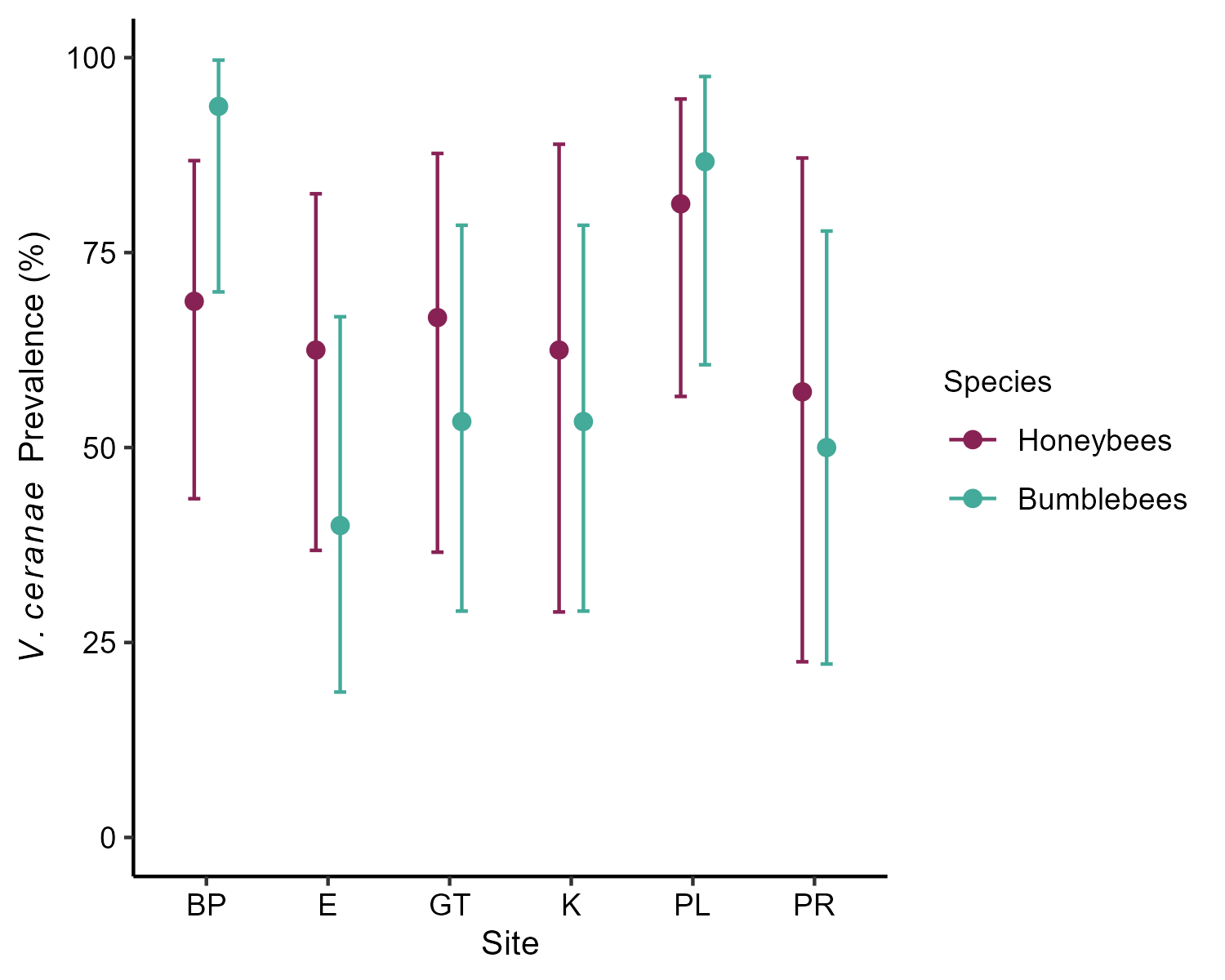
**

**Figure S2.** *V. ceranae* prevalence across the six field sites did not vary among honeybees but did vary in bumblebee hosts (Appendix S1: Table S10). The error bars signify a 95% confidence interval.

**
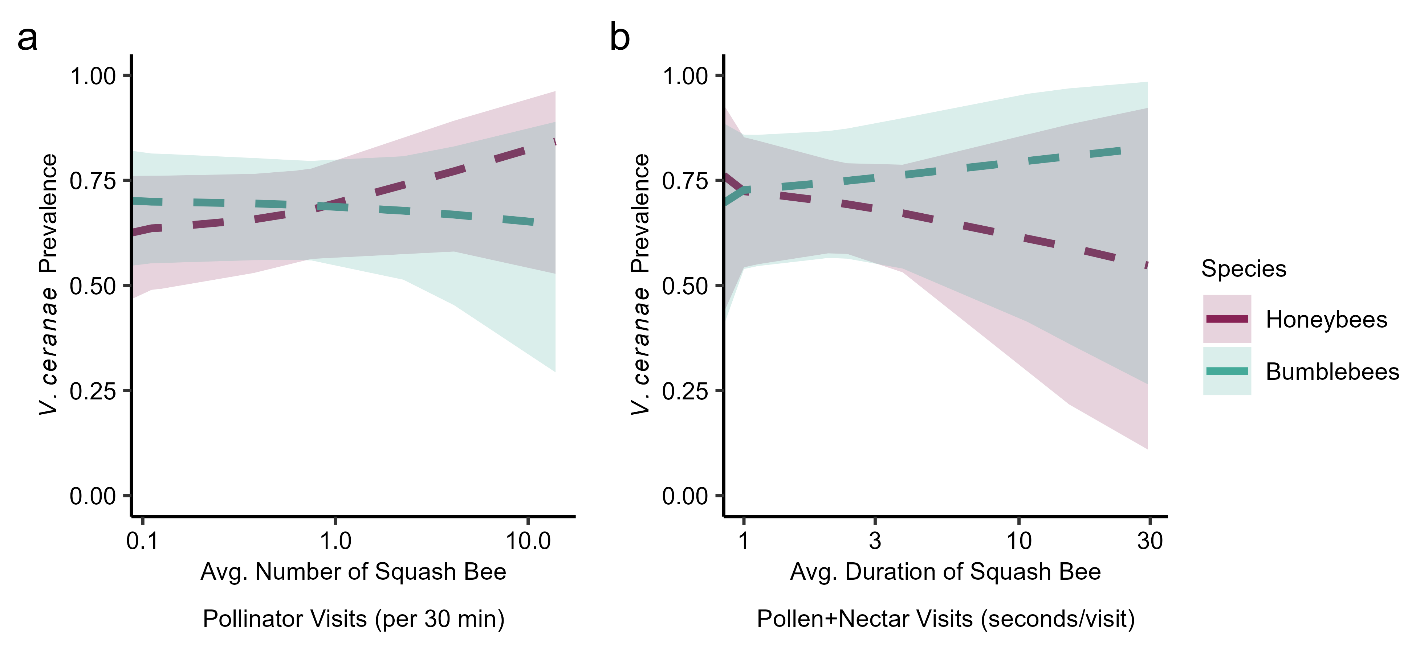
**

**Figure S3.** Neither the number of squash bee visits per 30 min nor the duration per visit to pollen + nectar impacted *V. ceranae* prevalence in honeybees or bumblebees. There was no change in *V. ceranae* prevalence in honeybees or bumblebees based on (a) average number of squash bee visits (per 30 min), or (b) average duration on pollen + nectar per visit (second/visit). Significant slopes are indicated by solid lines, while insignificant slopes are indicated by dotted lines. X-axes were converted to their original numerical values but are on a log scale, where zero values are on the y-axis.

**
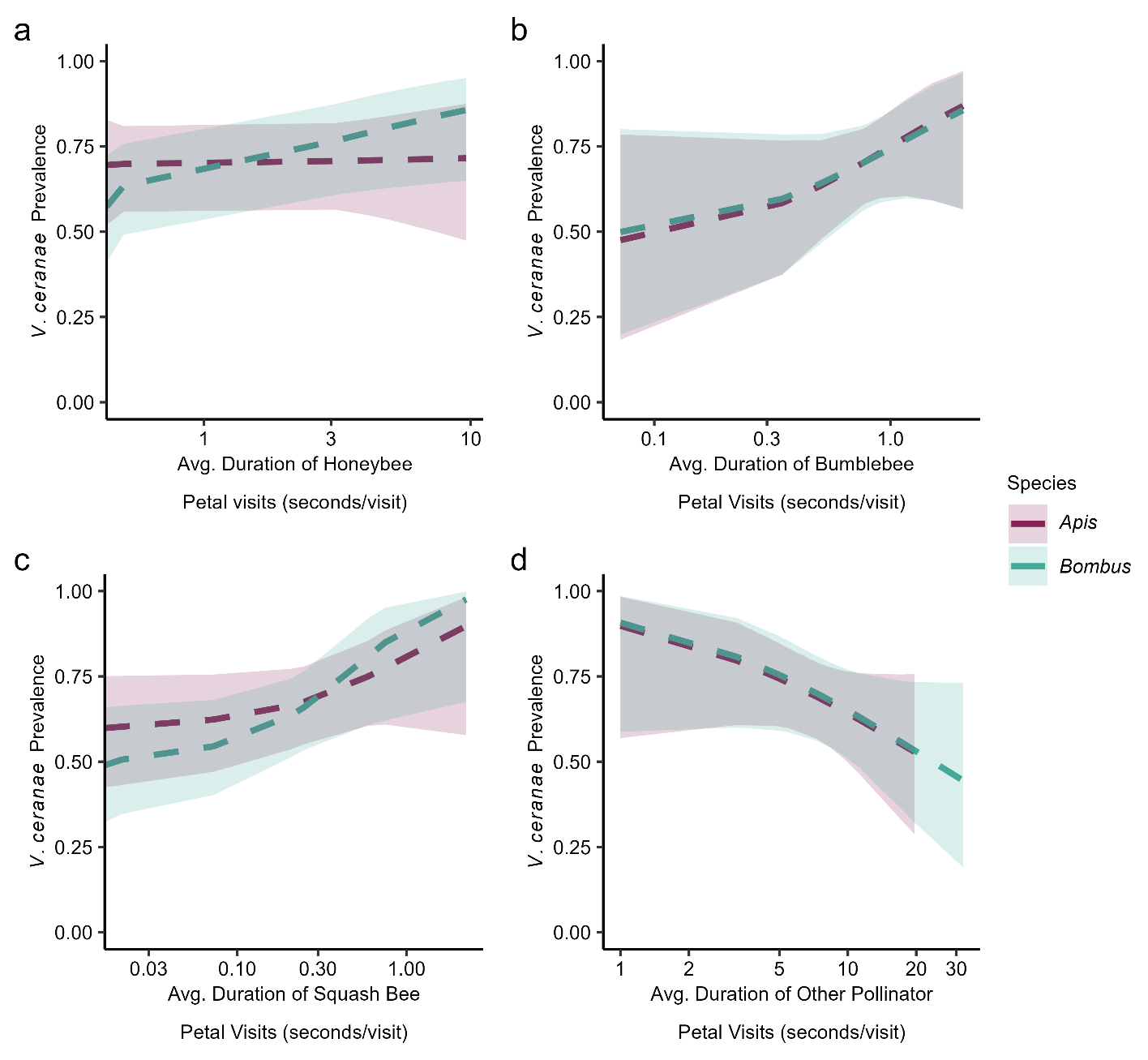
**

**Figure S4.** The duration per visit to petals by any pollinator taxa did not impact *V. ceranae* prevalence in honeybees or bumblebees. There was no change in *V. ceranae* prevalence in honeybees or bumblebees based on the average duration per visit on petals (second/visit) by (a) honeybees, (b) bumblebees, (c) squash bees, or (d) other pollinators. Significant slopes are indicated by solid lines, while insignificant slopes are indicated by dotted lines. X-axes were converted to their original numerical values but are on a log scale, where zero values are on the y-axis.

**APPENDIX S2**

**Trapping methodology**

To catch bees using the netting method, each transect was walked once for 30 minutes at approximately 08:00, 10:00, 11:00, and 12:00 before the squash flowers closed around midday. Any observed honeybees and bumblebees that were visiting squash flowers within 1.5 m of the transect were captured. To allow for maximum sample sizes, pan traps were used alongside netting. Brightly colored pan traps attract bees, who are subsequently trapped by soapy liquid in the pans and drown (Roulston et al. 2007). In our study, fluorescent blue, yellow, and white pan traps were placed in an alternating color pattern 5 m apart along each transect between the crop rows. Each trap was filled with a mixture of water and clear dish soap. Traps were set up by 07:00 am, checked every three hours for captured pollinators, and collected after the squash flowers closed around midday, for a total average duration of six hours.

**PCR procedure**

The PCR master mix contained 12.5 µL dH_2_O, 2 µL 10x buffer, 0.4 µL 10 mM dNTPs, 1 µL of each primer (10 mM), 2 µL 25 mM MgCl_2_, and 0.1 µL 5 U/µL Taq polymerase (Invitrogen, Carlsbad, CA, USA) per reaction. Reactions were run with an initial denaturation step at 94 ˚C for 2 min, 40 cycles containing denaturation at 94 ˚C for 30 s, annealing at 61 ˚C for 45 s, and extension at 72 ˚C for 2 min, followed by a final extension at 72 ˚C for 7 min and a cooling period at 10 ˚C for 2 min.

The PCR product was visualized on a 2% agarose gel by observing a 250 bp band. We extracted the 250 bp band with a High Pure PCR Product Purification and Gel Extraction kit (Roche, Basel, Switzerland) to clean the product for sequencing.

**Reference**

Roulston, T., Smith, S., & Brewster, A. (2007). A comparison of pan trap and intensive net sampling techniques for documenting a bee (Hymenoptera: Apiformes) Fauna. Journal of the Kansas Entomological Society, 80, 179–181.
